# Viniferin and its derivatives: a comprehensive review of structural variations and promising pharmacological applications in disease prevention and therapeutic development

**DOI:** 10.1007/s00210-025-04678-8

**Published:** 2025-11-29

**Authors:** Ahmed M. El-Dessouki, Kareem A. Attallah, Aya H. Eid, Eman S. Zaki, Samar S. Khalaf, Riham A. El-Shiekh, Nada M. Kamel, Rana M. ElBishbishy, Ahmed H. Elosaily

**Affiliations:** 1 Pharmacology and Toxicology Department, Faculty of Pharmacy6 of October City, Ahram Canadian University, Giza, 12566 Egypt; 2 Research and Development Department, Biotechnology Research Center, 23 July St., Industrial Zone, New Damietta, 34517 Egypt; 3 Pharmacology and Toxicology Department, Faculty of Pharmacy, Heliopolis University, Cairo, Egypt; 4 Biochemistry Department Faculty of Pharmacy, Heliopolis University, Cairo, Egypt; 5 Department of Pharmacognosy, Faculty of Pharmacy, Cairo University, Cairo, 11562 Egypt; 6 Department of Pharmacology and Toxicology, Faculty of Pharmacy, Cairo University, Kasr El-Aini Street, Cairo, 11562 Egypt; 7 Department of Pharmacognosy, Faculty of Pharmacy, Ahram Canadian University, Giza, 12573 Egypt; 8 Clinical Research Department, Damietta Directorate for Health Affairs, Egyptian Ministry of Health and Population, Damietta, Egypt

**Keywords:** Viniferin, Chemistry, Pharmacology, Health benefits, Functional foods, Drug discovery

## Abstract

Viniferin, a resveratrol-derived compound that belongs to a group of plant-produced stilbenoids, functions as a natural defense against microbial invasion, toxins, infections, and ultraviolet radiation. Alpha-(α-) viniferin (trimer), beta-(β-) viniferin (dimer), delta-(δ-) viniferin (oxidative dehydrodimer), epsilon-(ε-) viniferin (distinct dehydrodimer), gamma-(γ-) viniferin (isomeric oligomer), vitisin A (R-viniferin), and vitisin B (R2-viniferin) are structurally diverse forms with distinct pharmacological activities. Antioxidant studies showed that ε-viniferin exhibited a 2,2-diphenyl-1-picrylhydrazyl (DPPH) free radical scavenging half-maximal inhibitory concentration (IC₅₀) of about 80 µM. Also, suppression of nuclear factor kappa B, cyclooxygenase-2, and prostaglandin E₂ are anti-inflammatory mechanisms. R2-viniferin demonstrated an IC₅₀ of 9.7 µM against hepatocellular carcinoma HepG2 cells at 72 h, mediated through apoptosis and cell-cycle arrest, according to anticancer studies that demonstrated dose-dependent cytotoxicity. There have been reports of additional activity against models of glioblastoma and prostate cancer. In metabolic disorders, oral α-viniferin (20–40 mg/kg/day) improved lipid and glucose homeostasis in mice fed a high-fat diet, and it additionally improved liver and renal biomarkers such as blood urea nitrogen, creatinine, alanine aminotransferase, and aspartate aminotransaminase. Several bacterial strains have shown signs of preliminary antimicrobial action. By reducing excitotoxicity and oxidative stress, viniferins also have neuroprotective effects. They also have anti-melanogenic properties by blocking the tyrosinase and melanogenesis pathways. Collectively, viniferins demonstrate pleiotropic pharmacologic activities by defined molecular mechanisms and quantifiable dose-dependent effects. The properties classify viniferins as new multifunctional drug candidates for discovery and nutraceuticals, but they highlight the need for standardized pharmacologic assays, further preclinical validation, and pharmacokinetic optimization towards clinical use.

## Introduction

Known as phytoalexins, stilbenoids are phenolic compounds created by plants to defend themselves. These compounds are produced via environmental stress rather than metabolic synthesis. Examples of biotic stresses are pathogenic assaults, bacterial infections, and insect predators. However, the abiotic stresses include chemicals, ultraviolet radiation, and extreme temperatures. Stilbenoids, which are antioxidants, anti-inflammatory medications, and antibacterials, are synthesized by plants to protect themselves against environmental damage and illness (Akinwumi et al. 2018; González-Barrio et al. 2006; Houillé 2015; Ramawat and Mérillon 2013; Riviere et al. 2012). Grapevine (*Vitis vinifera*) is among the primary natural sources of these compounds (Fuloria et al. 2022). Structurally, stilbenoids possess a C6-C2-C6 framework and are categorized into five groups: stilbenes, oligostilbenes, bibenzyls, bisbibenzyls, and phenanthrenes. Resveratrol stands out as the most extensively studied stilbenoid, while viniferins are recognized as its oligomeric derivatives. Viniferin is a naturally occurring polyphenolic chemical that is formed upon resveratrol oxidation. Naturally, viniferin exists in three primary forms: alpha-, epsilon-, and delta-dimers, as well as alpha-viniferin, a trimer. Many other distinct types have been discovered, including R-viniferin (vitisin A), R2-viniferin (vitisin B), and β- and γ-viniferin (El Khawand 2018); Fuloria et al. ; Pawlus, 2012). These compounds play crucial roles in plant defence and have attracted interest for their potential therapeutic applications due to their varied bioactivities. This review is aimed at exploring viniferin’s chemical characteristics and pharmacological effects to assess its promise as a foundation for developing innovative therapeutic agents targeting various health conditions.

## Search strategy

To compile this review, extensive research was gathered from multiple scientific platforms such as Google Scholar, Scopus, and PubMed. The search employed specific terms including “viniferin” combined with “stilbenoid oligomers,” “*Vitis vinifera*,” and various study types like “in vitro,” “in vivo,” “biological studies,” “pharmacological studies,” “chemistry,” “toxicity studies,” and “pharmacokinetics” covering all relevant publications up to the search date. Articles not published in English or lacking abstracts were excluded.

## Natural sources

Viniferin, a resveratrol oligomer, exists in multiple structural forms including α-, ε-, δ-, ω-, R-, R2-, cis-, and trans-viniferin, each distributed across various plant species. α-Viniferin has been identified in numerous species, particularly in roots and stem barks, such as *Astilbe grandis* (Shi et al. 2021), *Caragana chamlagu* (Sim et al. 2014), *Caragana sinica* (Jeong et al. 2017), *Carex baccans* (Roy and Giri 2015b), *Carex humilis* (Chung et al. 2003b), *Cayratia trifolia* (Arora et al. 2009), *Dipterocarpus littoralis* (Lulan 2020), *Dipterocarpus verrucosus* (Zain et al. 2024), *Shorea roxburghii* (Moriyama, 2016), *Hopea exalata* (Ge et al. 2006), *Hopea ponga* (Sasikumar et al. 2019), *Iris clarkei* (Keckeis et al. 2000), *Shorea maxwelliana* (Zawawi et al. 2013), *Shorea ovalis* (Sutopo 2009), *Shorea seminis* (Aminah et al. 2002), *Vitis heyneana* (Ha et al. 2018), *Vitis quinquangularis* (Roat and Saraf 2020), and *Vitis vinifera* (Houillé 2015).

ε-Viniferin, the most widely studied form due to its notable biological activity, has been reported in *Bombax malabarica* (Lam et al. 2019), *Carex acuta*, *Carex lepidocarpa* (Tříska et al. 2024), *Cayratia trifolia* (Arora et al. 2009), *Cyphostemma crotalarioides* (Bala et al. 2000), *Dipterocarpus verrucosus* (Zain et al. 2024), *Dryobalanops lanceolata* (Ahmat et al. 2014), *Hopea parviflora* (Prabha et al. 2021), *Iris lactea* (Kim et al. 2020), *Paeonia lactiflora* (Yuk et al. 2013), *Paeonia ostii* (Tian et al. 2020), *Paeonia suffruticosa* (He et al. 2019)), *Parthenocissus quinquefolia* (Yang et al. 2014)), *Rheum lhasaense* (Liu et al. 2013), *Gnetum microcarpum* (Azmin et al. 2014), *Vatica lowii* King (Kamarozaman et al. 2025), *Vitis amurensis* (Kiselev et al. 2017), *Vitis labrusca* (Lambert et al. 2019), *Vitis rotundifolia* (Nopo-Olazabal et al. 2013), *Vitis thunbergii* (Cheng et al. 2020), and also extensively in *Vitis vinifera* (Houillé et al. 2015).

δ-Viniferin has been isolated from *Rheum undulatum* (Ha et al. 2018) and *Vitis labrusca* (Lambert et al. 2019), while both cis- and trans-ε-viniferin forms have been detected in *Cyphostemma crotalarioides* (Bala et al. 2000), *Paeonia lactiflora* (Yuk et al. 2013), *Paeonia ostii* (Tian et al. 2020), and *Paeonia suffruticosa* (He et al. 2019).

Additionally, R- and R2-viniferin (also known as vitisin A and B, respectively) are present in *Iris lactea* (Kim et al. 2020), *Vitis heyneana* (Ha et al. 2018), and *Vitis vinifera* (Houillé et al. 2015).

The rare ω-viniferin has only been reported in *Vitis vinifera*, which remains the most diverse natural source, producing nearly all major viniferin types including α-, ε-, δ-, ω-, trans-, R-, and R2-viniferin (Houillé et al. 2015).

## Chemical structure

Structurally complicated oligomers of resveratrol, viniferins are extraordinary natural polyphenols with therapeutic potential. Unlike its simpler ancestor, resveratrol, viniferins are formed by enzymatic oxidative coupling generating dimeric and trimeric molecules such as epsilon-viniferin, delta-viniferin, and alpha-viniferin. Though stretched into highly conjugated systems with several hydroxyl groups and fused ring structures, these compounds preserve the required stilbene backbone. The special property of viniferins is their stereochemical diversity. The two chiral centers often located at 7a and 8a of the dihydrofuran ring produced after dimerization mostly define this complexity. The spatial orientation of the hydrogen atoms, cis or trans configurations, produces four stereoisomers. Furthermore, E/Z isomerism of double bonds provides further structural variety (Fuloria et al. 2022).

The difficulty increases when differentiating between enantiomers like (+)- and (−)-ε-viniferin, which may display distinct biological activity. While optical rotation values ([α]D) provide some insight, the absence of conclusive crystallographic or chiroptical evidence frequently restricts researchers to designating only relative configurations. Consequently, several intricate oligomers containing viniferin are delineated with hypothesized geometries, either from biosynthetic inference or resemblance to previously defined analogs (Pawlus 2012).

Viniferins are oligomeric stilbenoids characterized by their intricate stereochemical diversity, mostly due to the presence of two chiral centers at positions 7a and 8a within the dihydrofuran ring generated during oxidative coupling. Four potential stereoisomers exist due to the chiral centers being able to adopt either a cis or trans configuration. Certain double bonds have E/Z isomerism, further enhancing structural diversity. This intricate spatial configuration is essential for modifying the biological effects of viniferins (Fuloria et al. 2022).

The stereochemistry of the primary viniferin isomers, namely, alpha (α), epsilon (ε), delta (δ), and the tetramers R (vitisin A) and R2 (vitisin B), substantially affects their interactions with biological targets. The trans-configuration, the predominant form of ε-viniferin, exhibits superior antioxidant and anti-inflammatory properties compared to the cis-isomers. Their structure has several hydroxyl groups and extended double bonds, enabling them to capture free radicals and alter the activity of enzymes essential for their therapeutic effects (Sy 2023).

Chemical modification of viniferin molecules, such as the addition of acetyl groups, has demonstrated enhanced efficacy in combating cancer. Enzymatic methylation and halogenation can improve their effectiveness against bacteria by modifying their interactions with microbial membranes and targeting essential enzymes. The combination of various viniferin isomers may produce synergistic effects, potentially enhancing their therapeutic efficacy beyond the constraints of individual molecules. This information regarding the structure–activity relationship illustrates the significance of stereochemical configuration and chemical functionalization in enhancing the efficacy of viniferin-based therapies (Fuloria et al. 2022).

## Isolation and spectroscopic data

The structural elucidation of viniferins requires a combination of isolation techniques and advanced spectroscopic tools, given their low natural abundance and tendency to co-occur with other polyphenols. The spectral spectrum of trans-ε-viniferin is thoroughly reported. In UV–Vis spectroscopy, distinct absorbance peaks are seen at 203, 230, and 324 nm in methanol, exhibiting a significant bathochromic shift to 211, 244, and 347 nm with the introduction of sodium hydroxide (Privat et al. 2002; Sahidin et al. 2017). The IR spectra verify the existence of phenolic hydroxyl groups (wide band at 3393 cm⁻^1^) and aromatic rings (bands at around 1606, 1513, and 832 cm⁻^1^).

The ^1^H-NMR spectra, obtained in deuterated acetone, reveals separate signals for aromatic protons on ring A (δ 7.21 and 6.83 ppm), a singlet at δ 6.24 ppm from ring B, and downfield doublets for ring C protons (δ 5.42 and 4.49%). The alkene and meta-coupled protons of rings D and E resonate within the range of δ 6.32–7.18 ppm, together defining a well-conjugated system.

The trimeric α-viniferin, with molecular formula C₄₂H₃₀O₉, has been characterized using field-desorption mass spectrometry, revealing a pseudomolecular ion at *m/z* 701 [M + Na]^+^ and a molecular ion at *m/z* 678 [M] + (Kitanaka et al. 1990). Its UV absorbance peaks at 285 nm, while IR spectra highlight typical polyphenolic features, including broad –OH stretching and aromatic C = C bands. In ^13^C-NMR, α-viniferin shows a total of 42 carbon signals, including six aliphatic methine carbons (δ 46.4–95.6 ppm), twelve aromatic CH groups, and eighteen quaternary carbons, many bearing oxygen substitutions. The ^1^H-NMR spectrum reveals pairs of doublets corresponding to methine groups and clearly separated signals for tetrasubstituted and 1,4-disubstituted aromatic rings. 2D NMR techniques, especially COSY and NOESY, provide vital spatial information. For example, NOE correlations between Ha, Hc, and Hr confirm trans configurations across the 2,3-dihydrobenzofuran units, solidifying the compound’s relative stereochemistry. Structural elucidation of δ-viniferin was accomplished using ^1^H-NMR and ^13^C-NMR in CD₃OD (Teng 2020). The proton spectrum spans δ 6.18 to 7.56 ppm, indicating aromatic and olefinic protons with diverse coupling patterns. Carbon signals in the δ 103–160 ppm range confirm a densely substituted aromatic framework. High-resolution mass spectrometry (HRESIMS) verified its molecular identity, with a precise ion at *m/z* 453.1336 [M + H]^+^, matching the theoretical mass for C₂₈H₂₁O₆.

## Biosynthesis

**Fig. 1:**
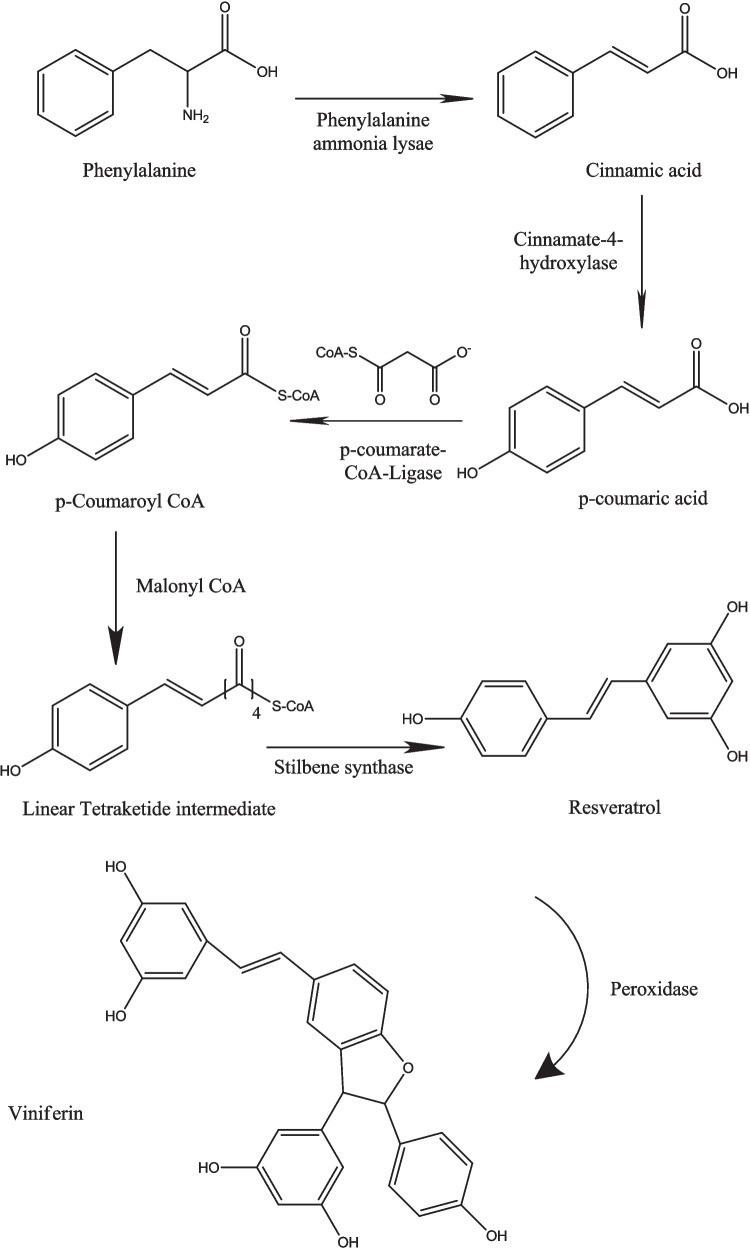
Viniferin biosynthesis pathway

The biosynthetic pathway of resveratrol in plants has been thoroughly characterized and involves a sequence of enzyme-mediated reactions. The process begins with the aromatic amino acid phenylalanine (Fig. 1), which undergoes deamination catalyzed by phenylalanine ammonia-lyase (PAL), resulting in the formation of cinnamic acid. This compound is subsequently hydroxylated to produce p-coumaric acid, followed by activation to p-coumaroyl-CoA via the action of CoA ligase. These transformations represent essential steps in the general phenylpropanoid pathway, which feeds into several classes of plant secondary metabolites.

The final and most defining step in resveratrol biosynthesis is catalyzed by stilbene synthase (STS). This enzyme orchestrates the condensation of p-coumaroyl-CoA with three units of malonyl-CoA, producing trans-resveratrol, a key phytoalexin involved in plant defence. Notably, STS competes directly with chalcone synthase (CHS), an enzyme involved in flavonoid biosynthesis, for the same substrates. Therefore, the regulation of STS activity is a major determinant of metabolic flux toward stilbene production, making it a central control point in the biosynthesis of resveratrol and its derivatives. While trans-resveratrol is a well-studied monomer, plants often extend its chemical versatility by producing oligomers, dimers, trimers, and higher-order structures through oxidative coupling mechanisms. This oligomerization is believed to occur non-enzymatically in planta, driven by the formation of phenoxyl radical intermediates.

Resveratrol oligomers are generated via the radical coupling of these oxidized phenolic species (Langcake and Pryce 1977). Upon oxidative activation, the reactive phenoxyl radicals couple at specific positions on the resveratrol backbone, yielding structurally distinct dimers. Two major coupling modes have been reported. The 8–10′ bond formation, which produces ε-viniferin, is a resveratrol dimer known for its strong antioxidant and antimicrobial properties, and 3–8′ coupling mode, which results in the formation of δ-viniferin, another prominent natural dimer with potential pharmacological applications. These regioisomeric linkages define the diversity of viniferin-type compounds observed in nature and suggest that plants employ oxidative oligomerization to enhance the structural complexity and bioactivity of their polyphenolic content.

## Quantification

Quantifying viniferins in biological samples or plant extracts is challenging due to their low concentrations, structural isomerism, and overlapping spectral features. Accurate analysis requires a combination of selective extraction, reference standards, and robust analytical platforms.

Initial identification often begins with UV–Vis spectrophotometry, useful for rapid screening thanks to the distinct absorbance peaks of viniferins. However, for accurate quantification, techniques like High-Performance Liquid Chromatography (HPLC) or Ultra-Performance Liquid Chromatography (UPLC) are the gold standard. These are typically paired with photodiode array (PDA) or mass spectrometric detection (LC–MS/MS) to improve specificity.

In such systems, compounds like α-viniferin can be reliably detected using their signature ions (e.g., *m/z* 701 [M + Na]^+^) (Kitanaka et al. 1990) and quantified by comparing peak areas to those of calibration curves generated from pure standards. Similarly, δ-viniferin can be quantified through its mass spectral profile (*m/z* 453.13) (Teng 2020) and unique chromatographic behavior. Proper method validation is essential for reproducibility, especially in complex matrices. These quantification strategies are critical for further research into the bioavailability, metabolism, and pharmacological evaluation of viniferins.

## Pharmacokinetics data

Primary pharmacokinetic (PK) studies reveal that viniferin isomers, particularly δ- and ε-viniferin, generally exhibit very low oral bioavailability (< 3%). This is attributed to a combination of poor absorption and extensive first-pass metabolism (glucuronidation and sulfation). Species differences in metabolism are notable; for instance, sulfation plays a larger role in ε-viniferin metabolism in vitro in humans compared to rats. Following systemic absorption (or IP injection), ε-viniferin shows a propensity to accumulate in adipose tissue, which may serve as a reservoir. Elimination occurs primarily via biliary excretion into the feces. Further research is needed to clarify the specific pharmacokinetic parameters of α-viniferin from primary sources and to fully elucidate the in vivo relevance of the observed adipose tissue accumulation. A comparative summary of available pharmacokinetic data in rats is presented in Table 1, covering oral and intraperitoneal routes and free versus encapsulated forms.

**Table 1:** Comparative PK data of viniferin in rats

Viniferin isomer	Route	Dose	Key PK observations	Reference
δ-Viniferin	Oral	70 mg/kg	Absolute oral bioavailability ≈ 2.3%; total absorption including metabolites ≈ 31.5%. ~ 60.3% excreted unchanged in feces; ~ 0.09% in urine	(Mao et al. 2016)
ε-Viniferin	Intraperitoneal	50 mg/kg	High AUC and long MRT in white adipose tissues compared to plasma, liver, and kidneys; metabolic conversion to glucuronides (major) and sulfates (minor); elimination mainly via feces	(Courtois et al. 2018)
ε-Viniferin (liposomal)	Oral	20 mg/kg	Encapsulation prolonged half-life of major glucuronide metabolite (εVG: 119 vs. 38 min free form), doubled plasma and tissue exposure	(Beaumont et al. 2021)

To overcome these pharmacokinetic limitations, nanotechnology-based delivery strategies have been explored. Encapsulation of ε-viniferin into multilamellar liposomes (MLL) improved its pharmacokinetic behavior in rats compared with free ε-viniferin. While encapsulation did not markedly alter plasma levels of the native compound, it significantly prolonged the half-life of its major glucuronide metabolite (εVG), increasing it almost three-fold (≈ 119 min vs. 38 min for the free form), and doubled plasma and tissue exposure to εVG. Moreover, encapsulated ε-viniferin led to higher concentrations in metabolically active tissues, including liver, kidneys, and adipose depots (Beaumont et al. 2021). These findings highlight that nanocarriers may enhance the stability and systemic persistence of viniferin metabolites, representing a promising avenue to overcome bioavailability barriers typical of plant-derived stilbenoids.

To date, human safety data on viniferins remain confined to topical use. Clinical investigations using α-viniferin-based formulations have consistently reported excellent dermal tolerance, with no signs of irritation or adverse reactions observed at concentrations up to 1000 µg/mL (Rahim et al. 2021). Likewise, studies on α-viniferin-containing preparations for managing hyperpigmentation have shown no cytotoxicity at clinically relevant doses (Yun et al. 2018).

However, a critical blind spot persists: the systemic safety profile of viniferins in humans is virtually unexplored. This represents a substantial gap in the scientific record, particularly as interest grows in expanding viniferin applications beyond dermatological contexts. As the compound continues to show promise in preclinical studies across a range of therapeutic areas, comprehensive toxicological evaluations in humans are urgently needed to guide future translational efforts and responsible clinical development.

## Pharmacological activities

### Antioxidant effects

Viniferins possess a variety of structural forms and biological activities. They are capable of scavenging nitric oxide (NO), hydroxyl (OH), and superoxide radicals (Skroza 2015). α-Viniferin, in particular, has been shown to enhance the DPPH•-scavenging rate in a dose-dependent manner. The mechanism of DPPH neutralization involves electron and proton (H⁺) transfer reactions (Burgos et al. 2020), which are primarily governed by redox processes. This implies that α-viniferin may participate in redox reactions to eliminate DPPH. Additionally, numerous studies suggest that antioxidant activity may also involve non-redox mechanisms (Li et al. 2016a).

One such non-redox pathway includes the chelation of transition metals, supported by the role of metals like Fe^2^⁺ in promoting reactive oxygen species (ROS) formation. For example, Fe^2^⁺ can facilitate the decomposition of H₂O₂ into OH radicals through the Fenton reaction. Thus, chelating Fe^2^⁺ can prevent the formation of OH radicals (Zhao et al. 2018).

Grapevine shoot extract (GSE) is a rich natural source of trans-ε-viniferin that is recognized for its potent antioxidant activity, primarily by boosting endogenous antioxidant defence systems (Weiskirchen and Weiskirchen 2016), and it has been associated with a variety of bioactive properties (Raj 2021), contributing to its high commercial value (Noviello 2022).

In addition to trans-ε-viniferin, GSE contains other resveratrol derivatives, including trans-viniferin, a dimeric form of resveratrol. Experimental findings show that isoproterenol (ISP) administration leads to an increase in malondialdehyde (MDA) levels, a marker of oxidative stress, which was significantly mitigated following GSE treatment (Feriani et al. 2017).

This reduction in MDA is attributed to the ROS-scavenging ability of GSE. Furthermore, rats pre-treated with GSE exhibited elevated levels of critical antioxidant enzymes such as catalase (CAT), superoxide dismutase (SOD), and glutathione (GSH), compared to ISP-only treated groups. Since GSH plays a vital role in intracellular antioxidant defense, its restoration supports the antioxidant potency of GSE (Sy 2023). This came in line with the study that reported about a comprehensive chemical profiling of *Vitis vinifera* extract has revealed a substantial presence of bioactive stilbenes, with ε-viniferin and R2-viniferin as the major constituents (Beaumont et al. 2022). Among these, ε-viniferin is the dominant compound, exhibiting notable antioxidant and anti-inflammatory properties. It has been shown to reduce cellular apoptosis, suppress ROS formation, and strengthen endogenous antioxidant defenses by upregulating key enzymes such as CAT and glutathione peroxidase (GPx) (Gómez-Zorita et al. 2023; Lim et al. 2025).

Additionally, ε-viniferin isolated from *V. vinifera* shoots using polyvinylidene fluoride (PVDF) membrane filtration and ethyl acetate partitioning has been found to activate sirtuin (SIRT) 3, a mitochondrial deacetylase. This activation leads to forkhead box O 3a (FOXO3) deacetylation, effectively reducing mitochondrial oxidative stress in models of Parkinson’s disease (PD). The compound’s free radical-scavenging efficacy has also been validated through DPPH and lipid peroxidation assays, supporting its broad-spectrum antioxidant potential (Ficarra 2016).

Comparative antioxidant assays have demonstrated that both ε-viniferin and δ-viniferins display superior activity in DPPH, FRAP, and NO scavenging tests compared to resveratrol and R-viniferin. Moreover, ε-viniferin has shown protective effects on vascular endothelial cells (VECs) under oxidative stress conditions. At specific concentrations, it was capable of preventing cell death, lowering ROS production, and enhancing the activity of antioxidant enzymes such as CAT and GPx in a time-dependent manner. Mechanistically, ε-viniferin exerts its free radical scavenging effects in UVB-irradiated human dermal fibroblasts (HDFs) through the suppression of matrix metalloproteinase-1 (MMP-1) expression, mediated by modulation of the extracellular signal-regulated kinase (ERK) and c-Jun N-terminal kinases (JNK) signaling pathways. Additionally, it promotes the restoration of Type I procollagen levels by activating the Smad signaling pathway and inhibiting ERK activity, indicating a multifaceted role in protecting skin cells from oxidative damage.

*Vitis vinifera* extract has demonstrated potential in promoting skin repair following UV-induced damage by mitigating oxidative stress. The pharmacological efficacy of the extract is largely attributed to its high content of eight active phytochemicals, with ε-viniferin and R2-viniferin being the most prominent contributors. Experimental findings indicate that *Vitis vinifera* extract provides protective effects against UVB-induced injury in human dermal fibroblasts (HDFs), primarily through its antioxidant and anti-inflammatory actions. These beneficial effects include the upregulation of Collagen Type I Alpha 1 Chain (COL1A1) and downregulation of MMP-1, suggesting its utility as a therapeutic candidate for alleviating UVB-induced skin damage. Mechanistically, these outcomes appear to be driven by the suppression of ERK signaling, which subsequently leads to the activation of the Smad pathway (Smad2/3/4) and inhibition of MMP-1 expression (Lim et al. 2025).

It has been postulated that δ-viniferin which is predominantly identified in UV-stressed grapevine foliage and certain wine varieties facilitates vascular endothelial repair primarily through NO-mediated mechanisms and exerts free radical-scavenging effects while attenuating hemoglobin oxidative degradation (Wu et al. 2020). Despite exhibiting moderate to high overall antioxidant capacity, the dehydro analog of δ-viniferin demonstrated only limited efficacy in suppressing lipopolysaccharide (LPS)-induced ROS generation. Nonetheless, dehydro-δ-viniferin exhibited a slightly superior inhibitory effect on LPS-induced oxidative stress compared to trans-δ-viniferin (Johnsen 2023).

**Fig. 2:**
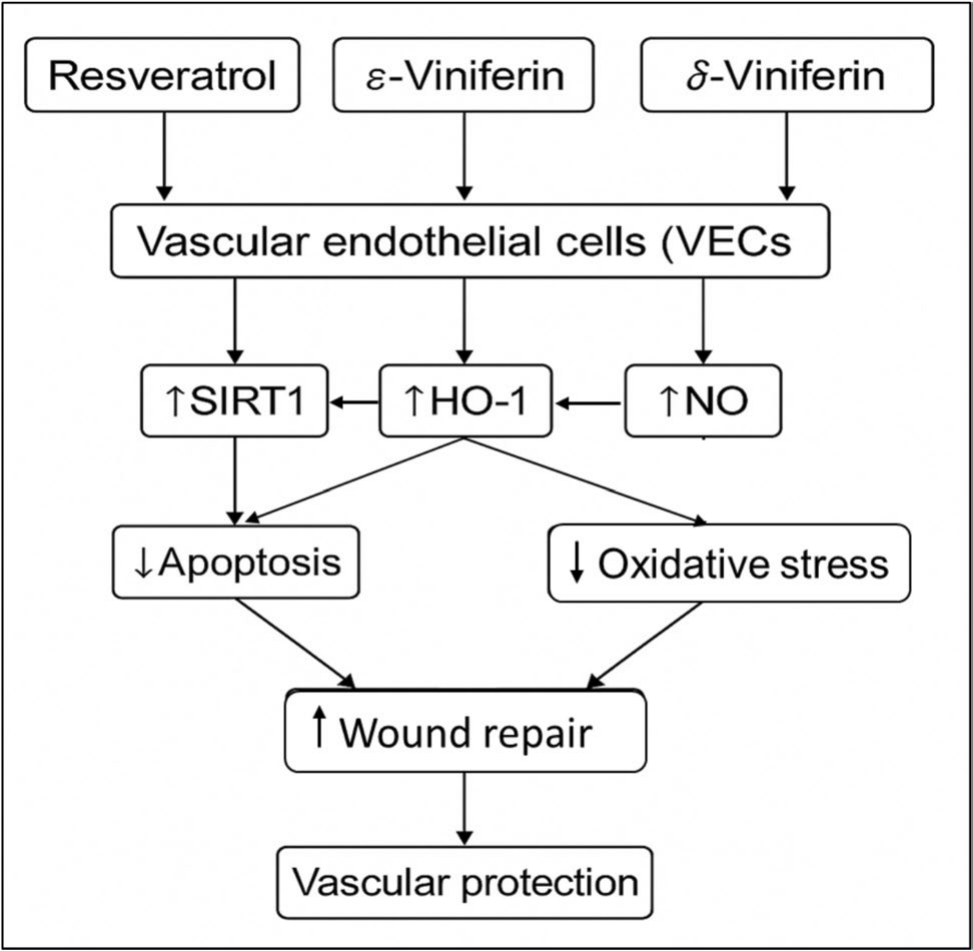
Schematic flow diagram represents vascular protective mechanisms of resveratrol and its dimeric forms ε-viniferin and δ-viniferin. HO-1, heme oxygenase-1; NO, nitric oxide; SIRT1, sirtuin 1; VECs, vascular endothelial cells

As shown in (Fig. 2), resveratrol and its dimeric forms ε-viniferin and δ-viniferin significantly promote endothelial wound repair through increasing NO production, upregulating SIRT1 and hemeoxygenase-1 (HO-1) expression. This lead to abating apoptosis and oxidative stress, consequently enhancing vascular protection and improving cell viability.

Numerous in vitro and in vivo studies have demonstrated the beneficial pharmacological and biological properties of R2-viniferen, notably its anti-inflammatory and antioxidant properties (Arruri, 2021; Giansanti, 2010; Maluchenko et al. 2021). R2-viniferin (vitisin A), a resveratrol-derived dimer composed of (+)-ε-viniferin and ampelopsin B (Bakrim, et al. 2025), exhibits antioxidant activity largely attributed to its chemical structure, which facilitates free radical scavenging and metal chelation. Its capacity to reduce Fe^2^⁺ to Fe^3^⁺ and neutralize ABTS and peroxyl radicals has been confirmed through assays such as FRAP, TEAC, and ORAC (Bakrim et al. 2025; García-Alonso et al. 2004).

A recent study by systematically compared the antioxidant properties of R-viniferin, ε-viniferin, and resveratrol using DPPH, NO-scavenging, and FRAP assays, revealing that ε-viniferin and R-viniferin exhibited relatively weak antioxidant effects. However, the combination of these stilbenes demonstrated synergistic activity in FRAP and NO assays, indicating potential interactive benefits despite low individual efficacy (Sy 2023).

### Anti-inflammatory effects

Stilbenoids have also demonstrated the ability to act on a broad range of cellular molecules involved in inflammatory signaling (Dvorakova and Premysl 2017). These compounds interact with cellular pathways by modulating transcription factors like NF-κB and nuclear factor erythroid 2-related factor 2 (Nrf2), influencing the expression of inflammatory mediators and antioxidant enzymes.

**Fig. 3:**
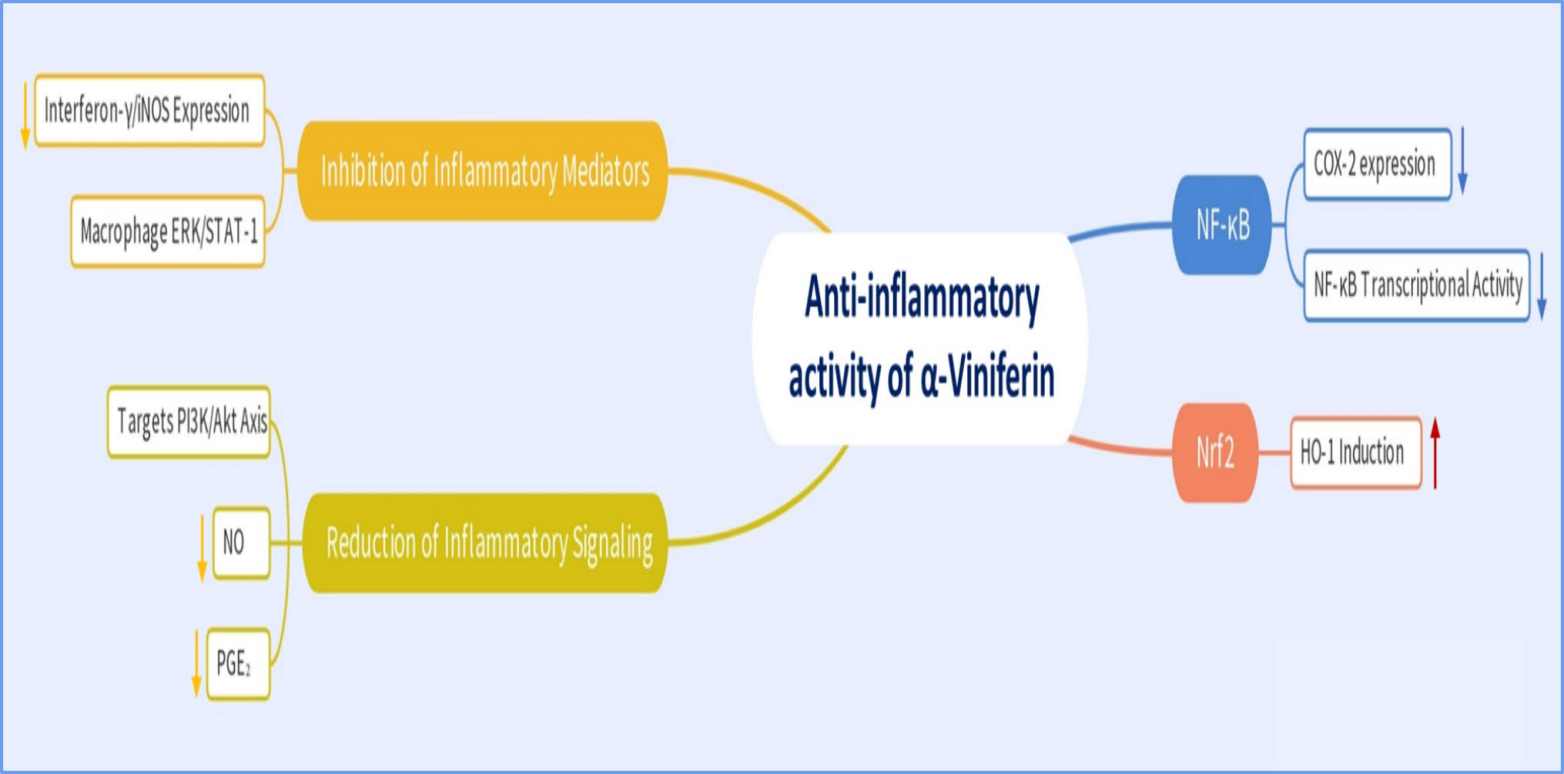
Schematic representation to the mechanistic anti-inflammatory activity of α-viniferin. Akt, protein kinase B; COX-2, cyclooxygenase-2; ERK, extracellular-signal-regulated kinase; HO-1, heme oxygenase-1; NF-κB, nuclear factor kappa-B; NO, nitric oxide; Nrf2, nuclear factor erythroid 2-related factor 2; PGE2, prostaglandin E2; PI3K, phosphoinositide 3-kinase; STAT-1, signal transducer and activator of transcription 1

Among the diverse oligostilbenoids isolated from *Vitis heyneana*, α-viniferin has emerged as the most potent in terms of inhibitory bioactivity (Fuloria et al. 2022). α-Viniferin has demonstrated significant anti-inflammatory properties across multiple studies (Dilshara 2014; Huang et al. 2023; Huang et al. 2021). Further evidence suggests that α-viniferin also attenuates inflammatory signaling by targeting the NF-κB pathway, specifically through inhibition of the phosphoinositide 3-kinase/protein kinase B (PI3K/Akt) axis, while simultaneously promoting the Nrf2-dependent induction of heme oxygenase-1 (HO-1). As represented in Fig. 3, this dual regulation leads to a marked reduction in both nitric oxide (NO) and prostaglandin E₂ (PGE₂) levels in microglial cells (Wu et al. 2020).

Moreover, in lipopolysaccharide (LPS)-stimulated models, α-viniferin significantly suppressed cyclooxygenase-2 (COX-2) expression and PGE₂ production, alongside a dose-dependent reduction in NO release. These anti-inflammatory effects were paralleled by effective inhibition of NF-κB transcriptional activity (Ha et al. 2018).

Esatbeyoglu et al. (2016) demonstrated that *Vitis vinifera* root extract, containing seven stilbenoids including R-viniferin and R2-viniferin, significantly inhibited the expression of inflammatory genes such as IL-1β and iNOS at the mRNA level in LPS-stimulated RAW264.7 murine macrophages. Furthermore, the extract conferred protection against H₂O₂-induced DNA damage, highlighting both its anti-inflammatory and antioxidant properties (Esatbeyoglu et al. 2016). Recent in vitro findings by Li (2023) expanded the scope of R2-viniferin’s immunomodulatory effects by demonstrating its capacity to suppress the formation of Ly6C^hi monocytes in cultured bone marrow-derived hematopoietic stem cells, implicating it in the regulation of both ulcerative colitis (UC) and atherosclerosis (Li 2023).

Similarly, a comparative study by Chang et al. (2017) revealed that R2-viniferin exerted moderate inhibition of NO in an NO-reduction assay, while ε-viniferin and RG-viniferin lacked notable activity (Chang et al. 2017).

In LPS-stimulated murine RAW 264.7 macrophages, δ-viniferin was shown to significantly suppress the expression of key pro-inflammatory mediators, including iNOS, COX-2, and phosphorylated inhibitor κBα (p-IκBα). These reductions coincided with a decrease in the downstream production of NO. Mechanistically, δ-viniferin’s inhibitory activity appears to involve multiple signaling pathways. It notably blocks the phosphorylation and degradation of IκBα, which prevents the subsequent activation of NF-κB, a central transcription factor in inflammation. Additionally, it reduces PI3K and Akt phosphorylation, further contributing to the downregulation of NF-κB signaling and transcriptional activity. These findings position δ-viniferin as a promising candidate for future development as an anti-inflammatory therapeutic agent (Hsieh 2016).

In a distinct context, dehydro-δ-viniferin, a structurally unique derivative, displayed differential immunomodulatory activity that depended on the bacterial stimulus. Specifically, while monomeric stilbenoids generally exert suppressive effects, dehydro-δ-viniferin notably enhances IL-12 production in response to *Lactobacillus acidophilus* stimulation. This is particularly relevant as IL-12 administration prior to MRSA pulmonary challenge in wild-type mice was shown to reduce bacterial burden and improve survival outcomes (Nguyen et al. 2015), highlighting its potential role in modulating host defense mechanisms.

**Fig. 4:**
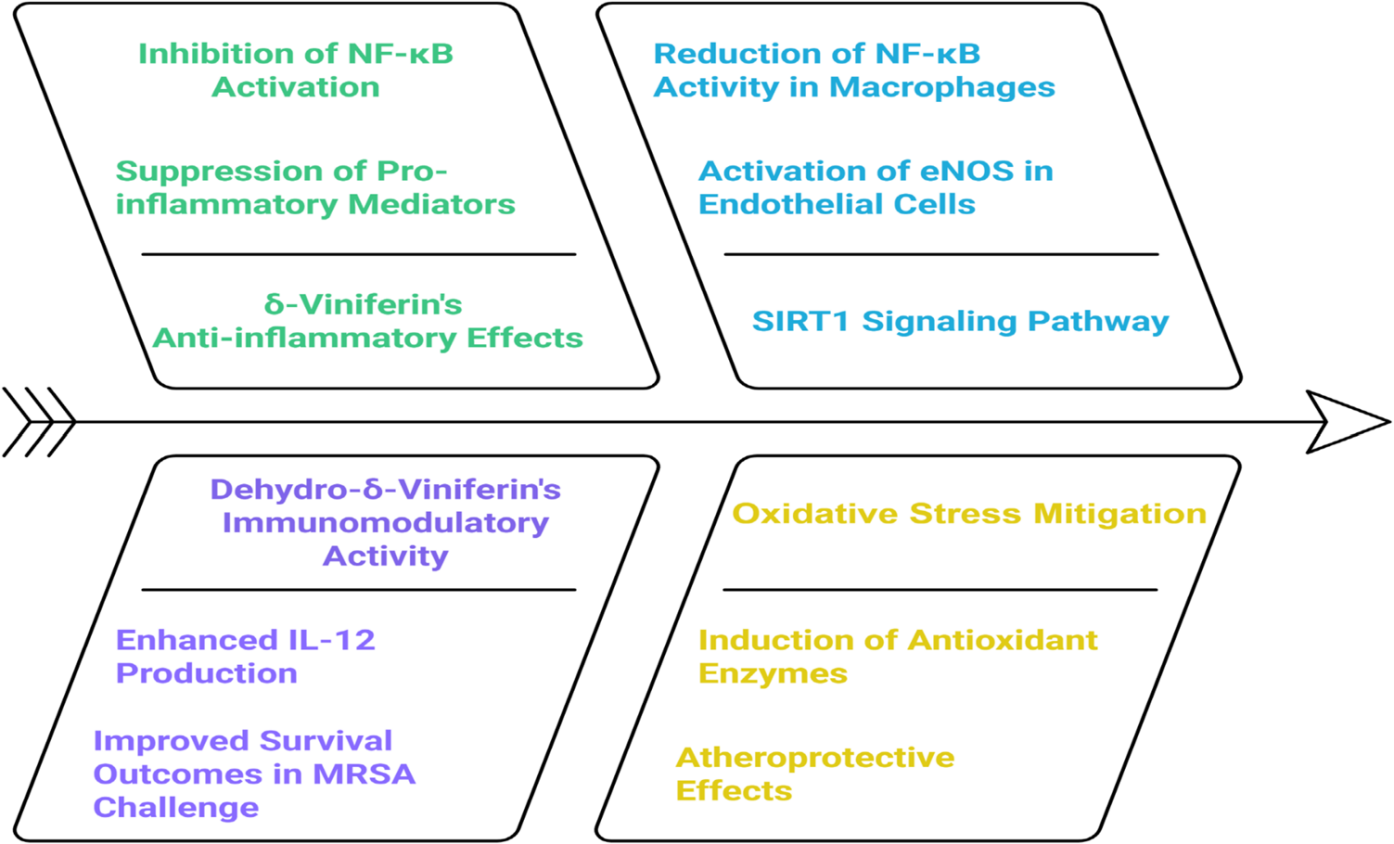
Anti-inflammatory mechanism differences between δ-viniferin and dehydro-δ-viniferin. eNOS, endothelial nitric oxide synthase; IL-12, interleukin-12; NF-κB, nuclear factor kappa-B; SIRT1, sirtuin 1

In parallel, the SIRT1 signaling pathway has been identified as a complementary target in inflammation and oxidative stress. SIRT1 activation enhances eNOS activity in endothelial cells, supporting vascular homeostasis. In macrophages, SIRT1 diminishes NF-κB activity, aligning with the anti-inflammatory profile observed with viniferin derivatives (Stein 2010). Moreover, SIRT1 mitigates oxidative stress by inducing antioxidant enzymes such as catalase and SOD in both astrocytes and endothelial cells (Cheng et al. 2014), thereby contributing to its overall atheroprotective effects (Fig. 4).

Recent findings highlight the efficacy of Vitis amurensis Rupr. (VA) extract, notably due to its high phenolic and flavonoid content. In UVB-irradiated human dermal fibroblasts (HDFs), VA extract containing ε-viniferin significantly suppressed IL-6 levels, suggesting its protective role against photo-induced inflammation (Lim et al. 2025).

Resveratrol, hopeaphenol, and (+)-ε-viniferin—as active agents—not only exhibited potent DPPH radical scavenging ability but also inhibited PGE₂ production in LPS-induced PHC-stimulated cells. In a rabbit model of acute inflammatory arthritis, administration of the extract significantly reduced serum PGE₂ and 18F-fluoro-2-deoxy-D-glucose (18F-FDG) levels, indicative of decreased inflammatory and metabolic activity. Focusing on α-viniferin, both oral (> 30 mg/kg) and intravenous (> 3 mg/kg) administration significantly attenuated carrageenan-induced paw edema in mice, reflecting systemic anti-inflammatory efficacy. This was associated with potent COX-2 inhibition, while its activity against COX-1 remained minimal, suggesting selective enzyme targeting (Chung et al. 2003). These results are in concordance with the work of Chung et al. (2010) who explored the action of α-viniferin in interferon gamma (IFN-γ)–stimulated macrophages. Their study revealed that α-viniferin suppresses ERK-mediated STAT-1 activation, consequently downregulating STAT-1-dependent inflammatory gene expression, a mechanism contributing to its broader immunosuppressive profile.

## Dual shield effect of viniferins as redox and cytokine modulators in key selected diseases

Viniferins combat obesity and metabolic dysregulation through their dual antioxidant and anti-inflammatory properties (Gómez-Zorita et al. 2023). As presented in Table 2, their anti-inflammatory effects involve inhibition of NF-κB signaling, which downregulates TNF-α and IL-1β, key mediators of chronic inflammation in obesity (Ohara et al. 2015). This suppression disrupts the crosstalk between adipocytes and macrophages, reducing adipose tissue inflammation and improving insulin sensitivity. For instance, ε-viniferin-enriched extracts mitigate hepatic steatosis and hyperlipidemia in obese models by lowering TNF-α-driven lipogenesis and promoting adiponectin secretion, which enhances glucose uptake in skeletal muscle (Gómez-Zorita et al. 2023). These mechanisms collectively improve metabolic homeostasis, positioning viniferins as promising nutraceuticals for obesity-related inflammation and insulin resistance (Ohara et al. 2015).

**Table 2:** Cytokine modulatory effect of viniferins is a key mediated seed in competing obesity and glucose homeostasis

Targeted diseases	Anti-obesity effect/mechanism	Which viniferin	Type of study	Description	Reference	Research gap	Clinical relevance
Obesity and glucose homeostasis	↓ Triglyceride accumulation in adipocytes	ε-Viniferin	In vitro	Reduces chemokine secretion from adipocytes	(Gómez-Zorita et al. 2023; Ohara et al. 2015)	Dose–response relationship not established; limited in vivo validation; long-term metabolic outcomes untested	Potential nutraceutical/adjunct for metabolic disorders
	↓ Pparγ expression	ε-Viniferin	In vitro	Downregulation of adipogenic gene at high doses			
	↓ Mcp1 expression	ε-Viniferin	In vitro and in vivo	Reduced inflammation markers in preadipocytes, adipocytes as TNFα			
	↓ Adipose tissue weights	ε-Viniferin	In vivo (mouse)	Reduced epididymal, retroperitoneal, and subcutaneous fat weights		Long-term metabolic/outcome data lacking; safety and PK in larger species/humans unknown	Potential nutraceutical with formulation/PK optimization required
	↓ Leptin expression	ε-Viniferin	In vivo	Decrease hormonal change associated with appetite and fat storage		Mechanistic linkage to appetite/energy balance needs validation in chronic models	May inform anti-obesity adjunct strategies
	↓ Intestinal glucose uptake (via SGLT1)	ε-Viniferin	In vitro (pig model)	Complete inhibition in BBMV from jejunum and ileum. Stronger than trans-resveratrol	(Guschlbauer et al. 2013)	Translation from ex vivo/in vitro to in vivo absorption and systemic effects unknown	Potential for post-prandial glycaemia modulation; needs formulation work
	↓ Plasma glucose and improved OGTT	ε-Viniferin	In vivo (rat and rat)	Improve insulin sensitivity and glucose tolerance	(Liu 2020)	Reproducibility across models and dose-range not established; no human data	Candidate for metabolic syndrome studies pending PK optimization
	↑ AMPK activation	ε-Viniferin	In vivo + in silico	Results in inhibition of lipogenesis, improving insulin resistance and suppressing appetite	(Liu 2020)	Downstream long-term metabolic consequences and safety unclear	Mechanistic support for anti-obesity potential; target for medicinal chemistry/formulation
	↓ α-Amylase activity	ε-Viniferin and trans-δ-viniferin	In vitro + in silico	Slowing starch digestion and postprandial glucose rise	(Mattio 2019b)	Limited in vivo confirmation; bioavailability/formulation issues	Could support dietary supplement approaches for glycemic control

Viniferins demonstrated significant therapeutic potential in neurodegenerative diseases by targeting oxidative stress and neuroinflammation, as evidenced by preclinical studies (Pislyagin 2025b; Sergi 2021). In PD cellular model using 6-hydroxydopamine (6-OHDA)-treated PC12 dopaminergic neurons, trans-ε-viniferin reduced oxidative stress-induced apoptosis by stabilizing mitochondrial integrity and suppressing caspase-3 activation. This effect was linked to its ability to scavenge ROS and enhance endogenous antioxidant defenses, such as glutathione, thereby restoring redox balance (Sergi 2021).

Further, in a rotenone-induced PD model using SH-SY5Y cells, ε-viniferin upregulated SIRT3, a mitochondrial deacetylase, which promoted FOXO3 deacetylation, reduced ROS production, and preserved mitochondrial function. This mechanism attenuated neuronal apoptosis and improved ATP synthesis, highlighting its role in mitochondrial homeostasis (Zhang et al. 2020).

**Fig. 5:**
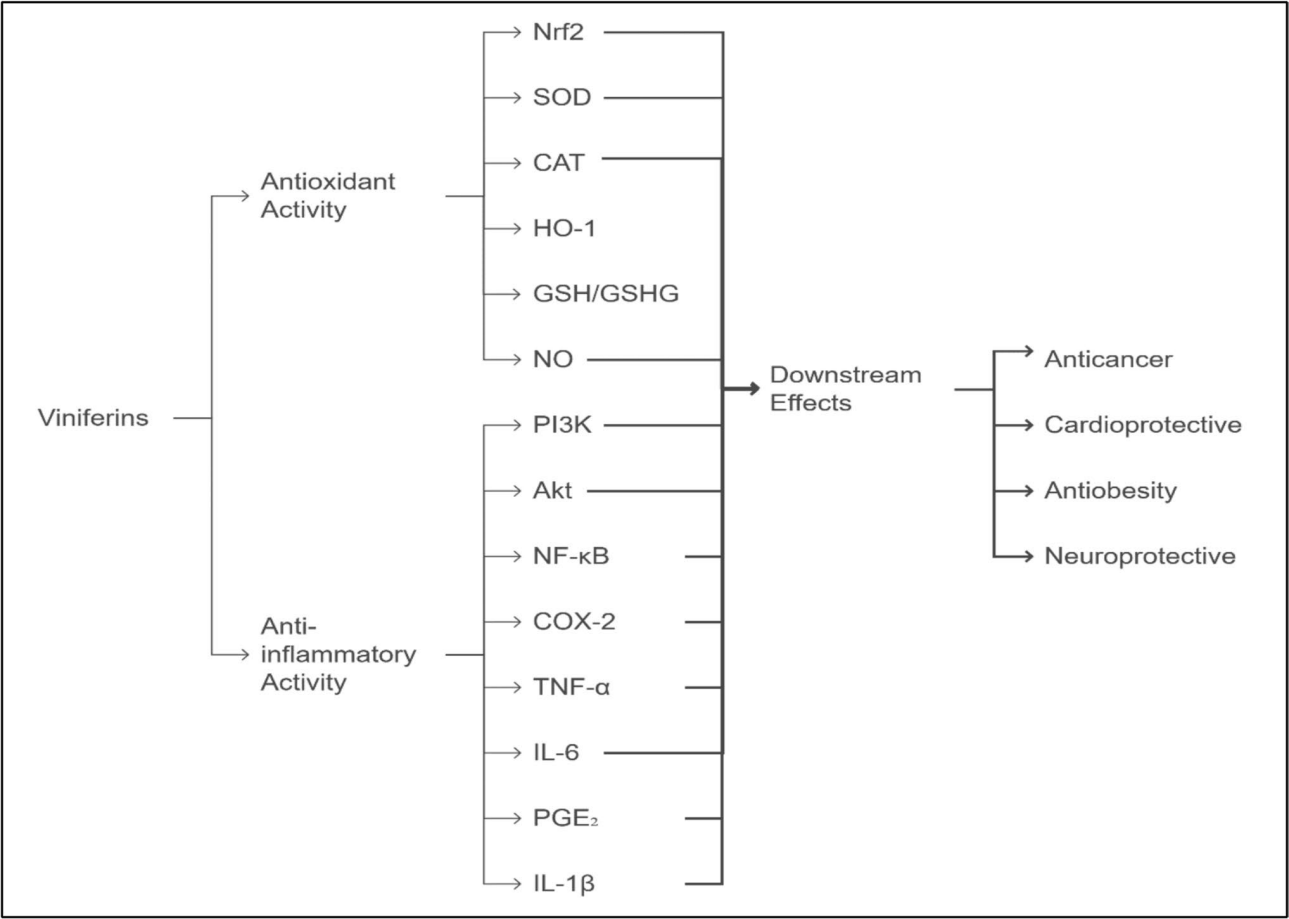
Flow chart assembles the anti-inflammatory and antioxidant targeted biomarkers and their downstream effects. Akt, protein kinase B; CAT, catalase; COX-2, cyclooxygenase-2; GSH, glutathione; HO-1, heme oxygenase-1; IL-1β, interleukin-1 beta; MDA, malondialdehyde; NF-κB, nuclear factor kappa B; NO, nitric oxide; Nrf2, nuclear factor erythroid 2–related factor 2; PGE₂, prostaglandin E₂; PI3K, phosphoinositide 3-kinase SOD, super oxide dismutase; TNF-α, tumor necrosis factor alpha

Similarly, in a neuron-glia co-culture PD model, ε-viniferin pre-treatment in microglia reduced LPS-induced neurotoxicity by lowering TNF-α and IL-6 release while synergizing with resveratrol to enhance anti-apoptotic Bcl-2 expression and suppress pro-inflammatory COX-2 (Sergi 2021). These findings underscore viniferins’ dual capacity to disrupt oxidative-inflammatory cascades, making them promising candidates for neurodegenerative therapy as summarized in Table 3. Collectively, the flow chart assembles the anti-inflammatory and antioxidant targeted biomarkers, and their downstream effects are displayed in Fig. 5.

**Table 3:** Redox and cytokine modulatory effect of viniferins are the key mediated seed in neurodegenerative diseases

Targeted diseases	Model/study type	Type of viniferin	Mechanism of action	Effect description	Reference	Research gap	Clinical relevance
Neurodegenerative diseases	In vitro (6-OHDA-treated PC12 dopaminergic neurons)	**trans-ε-Viniferin**	Scavenges ROS, stabilizes mitochondria, suppresses caspase-3 activation, enhances glutathione	Reduced oxidative stress**-**induced apoptosis, restored redox balance, protected neuronal viability	(Sergi 2021)	Lack of in vivo CNS efficacy and CNS PK data; dose–response not established	Candidate neuroprotective agent; requires CNS PK and in vivo efficacy testing
	In vitro (rotenone-induced SH-SY5Y cells)	**ε-Viniferin**	Upregulates SIRT3, promotes FOXO3 deacetylation, enhances ATP synthesis, reduces mitochondrial ROS	Improved mitochondrial homeostasis, reduced neuronal apoptosis, increased energy production	(Zhang et al. 2020)	No validated in vivo neurodegeneration models reported for translation	Candidate neuroprotective agent; requires CNS PK and in vivo efficacy testing
	In vitro (LPS-activated BV2 microglia)	**α-Viniferin**	Inhibits PI3K/Akt-NF-κB pathway; activates Nrf2/HO-1 signaling	↓ NO, ↓ PGE₂, ↓ TNF-α, ↓ IL-1β; **↑** antioxidant response; shifted microglial phenotype toward anti-inflammatory	(Dilshara 2014)	Mechanistic data strong in vitro but lacks in vivo corroboration and safety profiling	Potential immunomodulatory approach for neuroinflammation; needs animal model validation
	In vitro (neuron-glia co-culture PD model)	**ε-Viniferin**	Reduces LPS-induced neurotoxicity in microglia; synergizes with resveratrol to ↑ Bcl-2, ↓ COX-2, ↓ TNF-α, ↓ IL-6	Neuroprotection through combined anti-inflammatory and anti-apoptotic effects; microglial modulation enhances neuronal survival	(Sergi 2021)	Dose–response and chronic exposure effects not established; translation to PD in vivo models required	Candidate for combination therapies; further preclinical translational work needed

### Hepatoprotective effects

Viniferins, especially ε-viniferin, are oligomeric stilbenoids formed when resveratrol dimerizes. New studies in vitro and vivo support the idea that trans-resveratrol can help treat some liver diseases. For instance, it can make people who have had a liver donation much more likely to survive and reduce fat buildup, inflammation, and apoptosis. Conversely, several investigations indicated that viniferins may exhibit superior antioxidant properties compared to trans-resveratrol and might impede the generation of reactive oxygen species (Privat et al. 2002). Moreover, they have a variety of pharmacological actions, including anti-inflammatory, antioxidant, anti-fibrotic, and anticancer properties. Recent research have looked at their possible hepatoprotective benefits, particularly in the setting of liver damage caused by toxins like thioacetamide (TAA) (Fernandes et al. 2021). Viniferin’s hepatoprotective benefits are linked to numerous pathways, which include:

- Oxidative stress modulation: liver diseases often involve oxidative stress as a major contributor to hepatocyte damage (Cichoż-Lach and Michalak 2014). However, viniferin exerts potent antioxidant effects via several pathways.
  - ⚬ Upregulation of antioxidant enzymes: some recent studies revealed that viniferin enhances the activity of endogenous antioxidant enzymes. ε-Viniferin increases the expression and activity of enzymes such as SOD, CAT, GPx, and glutathione S-transferase (GST) levels to maintain redox balance (Fernandes et al. 2021).
  - ⚬ Regulation of Nrf2 pathway: viniferin activates Nrf2, which is a redox-sensitive transcription factor. Upon activation, it translocates to the nucleus and binds to antioxidant response elements (AREs) in the DNA (Joydip et al. 2016). This binding initiates the transcription of various cytoprotective genes, including those encoding for: HO-1, NAD(P)H quinone dehydrogenase 1 (NQO1), glutamate-cysteine ligase (GCL), glutathione S-transferases (GSTs), and superoxide dismutase (SOD). Activation of these genes enhances the cell’s ability to detoxify ROS and maintain redox homeostasis (Ngo and Duennwald 2022). This may lead to a-enhanced detoxification: upregulation of phase II detoxifying enzymes (Qader et al. 2020) and b-reduced oxidative damage: increased expression of antioxidant enzymes combating ROS (Carvalho et al. 2015).

- Anti-inflammatory effects: suppression of pro-inflammatory cytokines through Nrf2-mediated pathways (Mendonça 2024; Perez 2020). These effects could contribute to viniferin’s potential in preventing or mitigating diseases associated with oxidative stress, such as liver diseases. While direct evidence of viniferin activating Nrf2 pathway is limited, its monomeric counterpart, resveratrol, has been extensively studied in this context. Furthermore, another polyphenol, mangiferin, has demonstrated the ability to activate Nrf2, enhancing the antioxidant defense system in cells (Zhang et al. 2014). Given viniferin’s structural similarity to these compounds, it is plausible that it may exert comparable effects on the Nrf2 pathway. However, direct experimental evidence is needed to confirm this hypothesis. ε-Viniferin exerts its anti-inflammatory action in the liver primarily by interrupting NF-κB signaling cascade. By upregulating anti-inflammatory cytokines like IL-10 and downregulating pro-inflammatory cytokines like TNF-α and IL-6, viniferin diminishes inflammatory reactions in hepatic tissues. Mechanistically, ε-viniferin stabilizes the inhibitor IκBα and prevents phosphorylation-induced degradation, thereby blocking the p65 subunit’s translocation into the nucleus and consequent activation of pro-inflammatory genes (Al-Khayri et al. 2023).

In a thioacetamide-induced model of acute liver failure in Wistar rats, co-administration of ε-viniferin (5 mg/kg) together with resveratrol markedly suppressed hepatic TNF-α protein levels compared with untreated TAA controls. The same stilbene treatment also reduced IL-6 concentrations by approximately 30% demonstrating a significant down-modulation of this NF-κB-driven cytokine. Although direct measurements of IL-1β were not reported in that liver-failure study, inhibition of NF-κB signaling by ε-viniferin is expected to curtail IL-1β transcription; indeed, in BV2 microglial cells, ε-viniferin treatment significantly lowered IL-1β secretion following inflammatory stimulation (Al-Khayri 2023). Concomitantly, ε-viniferin shifts the balance toward resolution of inflammation by upregulating interleukin-10. In TAA-treated rats, combined resveratrol/ε-viniferin therapy doubled hepatic IL-10 levels relative to TAA alone, underscoring its capacity to enhance anti-inflammatory cytokine defenses. By suppressing NF-κB activation, ε-viniferin reduces the expression of iNOS and COX-2, thereby mitigating hepatic inflammation (Al-Khayri 2023; Fuloria et al. 2022).

- Antifibrotic action through inhibition of matrix metalloproteinases: viniferin has demonstrated hepatoprotective properties, particularly through its modulation of MMP-9, an enzyme implicated in extracellular matrix (ECM) degradation and liver fibrosis. Viniferin’s suppression of MMP-9 has a role in liver fibrosis. While ECM remodeling is essential for normal physiological processes, excessive MMP-9 activity can lead to pathological conditions like liver fibrosis by promoting ECM degradation and facilitating fibrogenic signaling pathways. Notably, MMP-9 can activate TGF-β, a cytokine that enhances fibrogenesis (Huang et al. 2021b).

A study investigating the combined effects of trans-resveratrol and ε-viniferin in a rat model of severe acute liver failure found that this combination significantly reduced MMP-9 expression. The downregulation of MMP-9 was associated with decreased oxidative stress and improved liver histology, suggesting a protective effect against liver injury (Fernandes 2021). While the exact molecular pathways through which viniferin suppresses MMP-9 expression are still under investigation, insights can be drawn from studies on resveratrol, its monomeric counterpart. Resveratrol has been shown to inhibit MMP-9 expression by modulating signaling pathways such as MAPKs and NF-κB (Behl et al. 2021; Xu et al. 2018).

Given the structural similarities between resveratrol and viniferin, it is plausible that viniferin may exert its effects through similar mechanisms, leading to the suppression of MMP-9 and attenuation of fibrogenic processes. The ability of viniferin to modulate MMP-9 expression positions it as a potential therapeutic agent in the management of liver fibrosis. By suppressing MMP-9, viniferin may help maintain ECM integrity, prevent excessive fibrotic tissue formation, and preserve liver function (Kim et al. 2015). To sum up, viniferin downregulates MMP-9**,** reducing ECM degradation and inhibiting fibrogenesis. It also modulates tissue inhibitor of metalloproteinase-1 (TIMP-1) levels to maintain ECM homeostasis (Liang 2023). However, further research is necessary to fully elucidate its mechanisms of action and to evaluate its efficacy and safety in clinical settings.

**Fig. 6:**
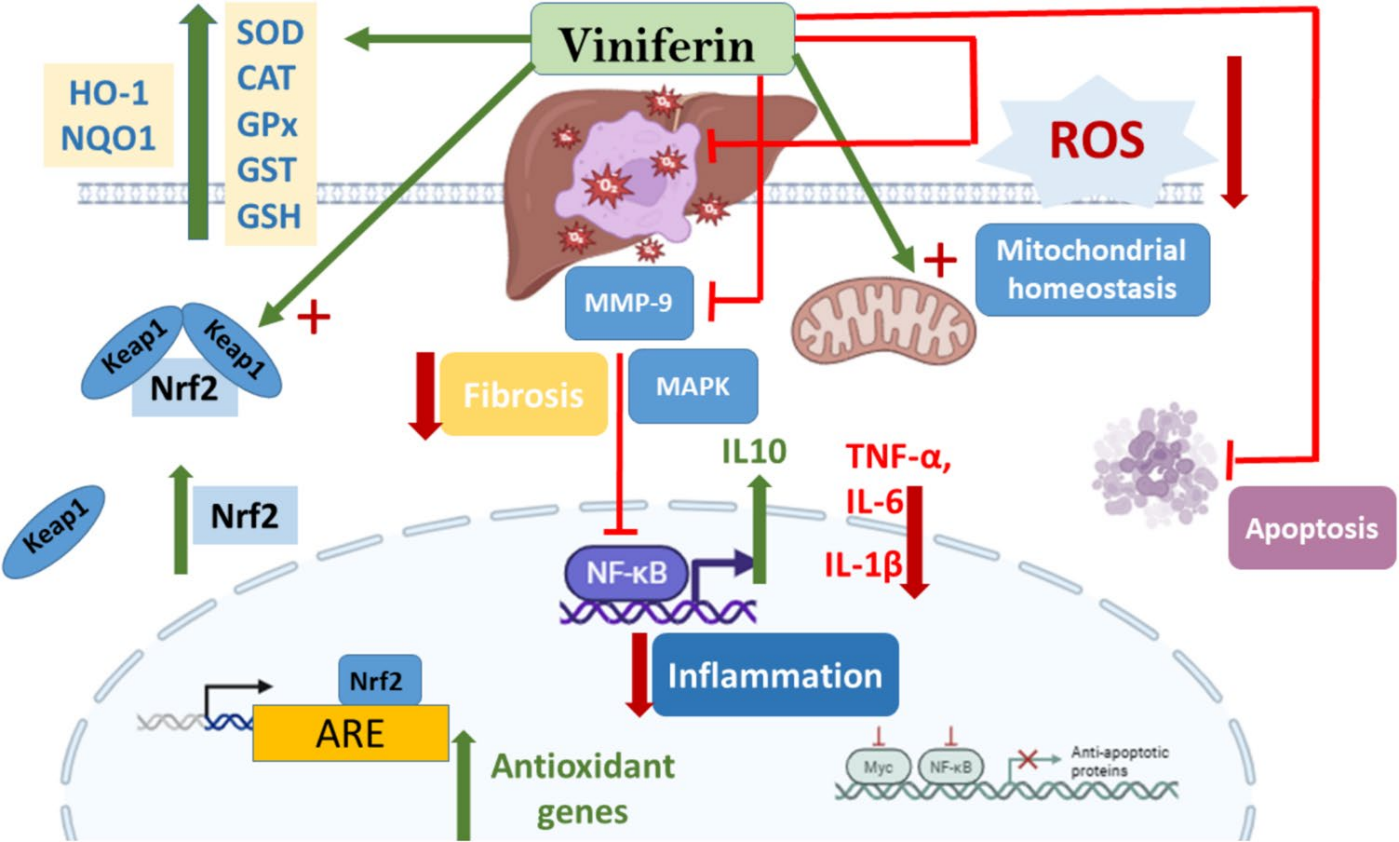
Viniferin possible hepatoprotective targets. CAT, catalase; GPx, glutathione peroxidase; GSH, glutathione; GST, glutathione S-transferase; HO-1, heme oxygenase-1; IL-1β, interleukin-1 beta; IL-6, interleukin-6; IL-10, interleukin-10; Keap1, Kelch-like ECH-associated protein 1; MAPK, mitogen-activated protein kinase; MMP-9, matrix metalloproteinase-9; NF-κB, nuclear factor kappa-B; NQO1, NAD(P)H quinone oxidoreductase 1; Nrf2, nuclear factor erythroid 2-related factor 2; ROS, reactive oxygen species; SOD, superoxide dismutase; TNF-α, tumor necrosis factor alpha

- Mitochondrial protection: viniferin improves mitochondrial membrane potential and prevents mitochondrial swelling**,** preserving ATP production and reducing hepatocyte apoptosis under stress conditions. ε-Viniferin fortifies mitochondrial function in hepatocytes by preserving the electrochemical gradient across the inner membrane and averting osmotic swelling of the matrix. By inhibiting the opening of the mitochondrial permeability transition pore, ε-viniferin prevents rapid influx of solutes and water into the matrix, thereby maintaining cristae integrity and sustaining ATP synthesis via oxidative phosphorylation (Zhang et al. 2022). This preservation of bioenergetic capacity limits cytochrome c release and downstream caspase-3 activation, resulting in markedly reduced hepatocyte apoptosis under chemical or oxidative stress.
- Apoptosis regulation: ε-viniferin shifts the balance of hepatocyte apoptotic regulators toward cell survival by upregulating the anti-apoptotic protein B cell lymphoma 2 (Bcl-2) while downregulating pro-apoptotic (Bcl-2)-associated X (Bax) and limiting caspase-3 activation. In hepatocellular carcinoma (HepG2) cells treated with 98.3 µM ε-viniferin, Bcl-2 mRNA levels rose to 289% of control at 24 h (*p* < 0.05), reflecting a strong induction of anti-apoptotic signaling (Özdemir et al. 2016). In the same cells and time point, Bax transcripts fell to just 14% of control values, indicating pronounced suppression of a key pro-apoptotic effector. Correspondingly, caspase-3 (pro-apoptotic proteins) activity in ε-viniferin-treated hepatocytes was measured at only 7.1% following 24 h exposure, compared with significantly higher activation in untreated or vincristine-only groups, demonstrating effective inhibition of the execution phase of apoptosis. Together, these changes reinforce mitochondrial integrity and reduce hepatocyte necrosis under stress conditions (Özdemir et al. 2016). To sum up, ε-viniferin exhibits robust hepatoprotective properties by targeting oxidative stress, inflammation, fibrosis, mitochondrial function, and apoptotic pathways. Its ability to modulate Nrf2, NF-κB, and MMP signaling underscores its potential in therapeutic strategies for liver injury and fibrosis (Fig. 6). Further research, especially clinical trials, is essential to confirm its efficacy and safety in human populations.

### Antidiabetic effects

Viniferin has attracted significant attention for its pharmacological properties, including antidiabetic potential. Among its various isomers, ε-viniferin and α-viniferin have been the most studied for their biological activities. Among its bioactivities, its antidiabetic effects have garnered growing interest. These effects involve multiple molecular targets and signaling pathways associated with glucose homeostasis, insulin signaling, oxidative stress, and inflammation.

- Mechanism of action in glucose metabolism:
  - ⚬ Activation of AMPK pathway: viniferin has been reported to modulate glucose metabolism by enhancing insulin sensitivity and glucose uptake in peripheral tissues. ε-Viniferin has been shown to activate AMPK pathway which plays a pivotal role in regulating cellular energy metabolism and homeostasis (Liu et al. 2020). ε-Viniferin has been shown to activate AMPK in skeletal muscle and liver tissues, leading to promoting glucose uptake, fatty acid oxidation, and inhibiting hepatic gluconeogenesis (Hung et al. 2019). Mechanistically, AMPK activation promotes the translocation of glucose transporter type 4 (GLUT4) to the plasma membrane in muscle cells, enhancing glucose uptake. It also inhibits the expression of phosphoenolpyruvate carboxykinase (PEPCK) and glucose-6-phosphatase (G6Pase), key enzymes in gluconeogenesis (Hardie et al. 2012).

- Inhibition of α-glucosidase and α**-**amylase enzymes: viniferin exhibits competitive inhibition of α-glucosidase and α-amylase, key digestive enzymes responsible for carbohydrate hydrolysis (Mattio et al. 2019b; Sasikumar et al. 2019). This action delays glucose absorption in the intestines, thereby attenuating postprandial hyperglycemia, a hallmark of type 2 diabetes. Viniferin’s IC₅₀ values for α-glucosidase inhibition have been reported to be comparable to or even lower than acarbose, a clinically used inhibitor (Lulan 2020).
  - ⚬ Effects on insulin secretion and pancreatic β-cell protection: in pancreatic β-cells, viniferin exhibits protective effects largely by counteracting oxidative stress-induced apoptosis, a major contributor to β-cell dysfunction in diabetes. A study in INS-1 pancreatic β-cell lines and streptozotocin (STZ)-induced diabetic rats demonstrated that viniferin treatment significantly increased insulin secretion via upregulation of PI3K/Akt signaling pathway, indicating a potential role in β-cell preservation and function (Alhajje et al. 2024; Martiniakova 2025). Additionally, viniferin improved the expression of Bcl-2 and suppressed caspase-3 and Bax, preserving β-cell mass (Peng et al. 2024). Moreover, viniferin has demonstrated antioxidant properties by upregulating endogenous antioxidant enzymes like SOD and CAT, further protecting pancreatic tissues from oxidative damage (Fernandes et al. 2021; Zhao et al. 2016).

- Anti-inflammatory effects related to insulin resistance via NF-κB and JNK inhibition: insulin resistance is often linked to chronic low-grade inflammation, mediated by pro-inflammatory cytokines. Viniferin has been found to inhibit the NF-κB and JNK signaling pathways, reducing the expression of inflammatory mediators such as TNF-α, IL-6 and iNOS in adipose tissue and liver, thereby improving insulin signaling pathways (Dilshara 2014). In high-fat diet-induced diabetic mice, viniferin administration led to reduced inflammatory infiltration and improved insulin sensitivity, suggesting its dual action as an anti-inflammatory and insulin-sensitizing agent (Laurindo 2023); Ren et al. 2019; Shazmeen et al. 2021).
- Antioxidant enzyme upregulation improving insulin signaling: viniferin possesses potent antioxidant properties, reducing ROS accumulation in pancreatic and peripheral tissues. It upregulates the expression and activity of SOD, CAT, and GPx, contributing to cellular protection and improved insulin signaling (Beaumont 2022; Gómez-Zorita 2023). This antioxidant effect also stabilizes mitochondrial membranes, preventing the release of apoptogenic factors in β-cells and other insulin-sensitive tissues (Papuc 2022).
- In vivo evidence and clinical perspectives: in vivo studies reinforce viniferin’s antidiabetic effects. Oral administration of viniferin in diabetic rodent models resulted in significantly decreased fasting blood glucose levels and improved lipid profiles (Liu et al. 2020). Moreover, in high-fat diet (HFD)-induced diabetic mice, viniferin administration (10–50 mg/kg/day) significantly reduced fasting blood glucose, HbA1c levels, and insulin resistance indices (HOMA-IR) (Martiniakova 2025). It also improved hepatic lipid profiles**,** suggesting systemic metabolic improvement. However, despite promising preclinical data, there is a lack of well-controlled human clinical trials, which limits the translational potential of viniferin for diabetes therapy at this stage. Nevertheless, its favorable safety profile, natural abundance, and multi-target mechanisms make viniferin a promising candidate for adjunct therapy in type 2 diabetes mellitus (T2DM) management (Duta-Bratu 2023).

**Fig. 7:**
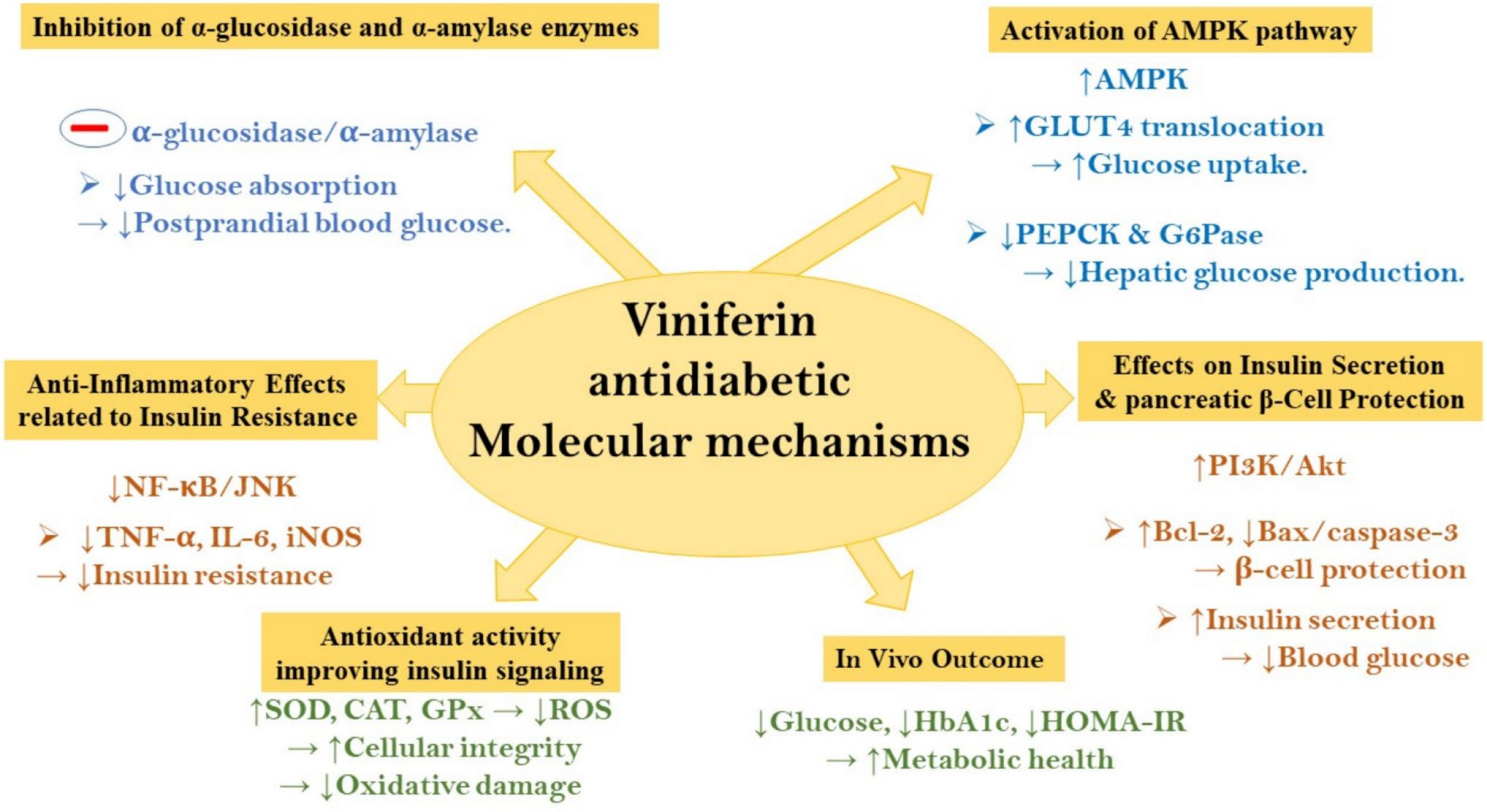
Schematic diagram summarizes viniferin antidiabetic molecular mechanisms. AMPK, adenosine monophosphate-activated protein kinase; Akt, protein kinase B; Bax, Bcl-2-associated X protein; Bcl-2, B cell lymphoma 2; CAT, catalase; G6Pase, glucose-6 phosphatase; GLUT4, glucose transporter type 4; GPx, glutathione peroxidase; HbA1c, hemoglobin A1c; HOMA-IR, homeostatic model assessment for insulin resistance; iNOS, inducible nitric oxide synthase; IL-1β, interleukin-1 beta; IL-6, interleukin-6; IL-10, interleukin-10; JNK, c-Jun N-terminal kinase; NF-κB, nuclear factor kappa-B; MAPK, mitogen-activated protein kinase; MMP-9, matrix metalloproteinase-9; PEPCK, phosphoenolpyruvate carboxykinase; PI3K, phosphoinositide 3-kinase; ROS, reactive oxygen species; SOD, superoxide dismutase; TNF-α, tumor necrosis factor alpha

These mechanisms collectively position viniferin as a promising candidate for adjunctive therapy in T2DM (Fig. 7). However, despite compelling preclinical evidence, clinical trials are necessary to validate its efficacy and safety in humans.

### Central nervous system (CNS) effect

Viniferin has garnered considerable attention for its potent biological activity in the central nervous system (CNS). Its complex structure, enhanced stability, and improved pharmacokinetic properties compared to monomeric resveratrol derivatives render it a highly promising compound for the treatment and management of a variety of CNS disorders. Numerous preclinical studies have demonstrated that viniferin exerts neuroprotective, anti-inflammatory, antioxidant, and neuromodulatory effects through the modulation of diverse molecular and cellular targets (Naia et al. 2021; Sergi, 2021). This section explores viniferin’s therapeutic efficacy across a wide range of CNS pathologies, categorized according to its mechanistic and disease-specific contributions.

Viniferin, with ε-viniferin being the most studied isomer, demonstrates potent anti-amyloidogenic effects that are central to its protective role in Alzheimer’s disease (AD). It impedes the aggregation and oligomerization of β-amyloid (Aβ) peptides by directly interacting with their hydrophobic regions, thereby destabilizing toxic fibrils and facilitating the clearance of Aβ from neuronal environments. This interaction reduces synaptic dysfunction, neurotoxicity, and cognitive impairment in experimental AD models. Furthermore, viniferin reduces tau hyperphosphorylation and enhances cholinergic neurotransmission, mitigating neurotoxicity and cognitive decline in Alzheimer’s models (Caillaud et al. 2019; Freyssin et al. 2022).

In PD, characterized by the progressive loss of dopaminergic neurons in the substantia nigra, viniferin offers neuroprotection by curbing α-synuclein aggregation and improving mitochondrial health. It stabilizes mitochondrial membrane potential, prevents ATP depletion, and inhibits apoptosis-inducing factors such as caspase-3 and cytochrome c. By preserving the functional integrity of dopaminergic circuits, viniferin delays motor deficits and sustains dopaminergic neurotransmission, thereby contributing to better clinical outcomes in PD models (Banerjee et al. 2024; Zhang et al. 2020).

Chronic neuroinflammation, driven primarily by sustained activation of microglia and astrocytes, contributes to the pathology of numerous CNS disorders (Kwon and Koh 2020; Singh 2022). Viniferin influences immune regulation by shifting the microglial phenotype from a pro-inflammatory M1 state toward an anti-inflammatory M2 state. It suppresses the NF-κB signaling cascade, thereby decreasing the production of inflammatory cytokines such as TNF-α, IL-6, and IL-1β. Simultaneously, it enhances the release of IL-10 and other neuroprotective mediators, fostering a microenvironment that supports neuronal survival and tissue repair (Banerjee et al. (Banerjee, 2024; Sergi 2021).

In addition to immunomodulation, viniferin activates the Nrf2/ARE pathway, leading to increased expression of antioxidant enzymes including HO-1, GPx, and SOD. This mitigates oxidative damage resulting from ROS accumulation. The combined antioxidant and anti-inflammatory effects make viniferin particularly effective in preventing the neurodegenerative cascade and in halting the progression of inflammatory CNS conditions (Dilshara 2014; Pislyagin 2025).

Viniferin reduces ischemia-induced neuronal apoptosis by modulating key apoptotic regulators, including upregulating Bcl-2 and downregulating Bax and cleaved caspase-3. It supports mitochondrial biogenesis, preserves ATP production, and maintains redox balance in neurons exposed to ischemic stress. Additionally, viniferin’s anti-inflammatory actions in post-ischemic tissue promote faster functional recovery and improved neurological outcomes (Beaumont 2022; Kim 2012). Viniferin displays notable anticonvulsant effects in experimental seizure models by targeting multiple excitatory and inhibitory signaling pathways. One of its principal actions is the inhibition of glutamate-induced excitotoxicity via modulation of N-methyl-D-aspartate receptor (NMDA) activity, thereby preventing overstimulation of excitatory neuronal circuits. It concurrently attenuates calcium influx and oxidative damage in hippocampal neurons, reducing neuronal stress and the risk of seizure-induced neurodegeneration. Furthermore, viniferin enhances GABA neurotransmission, which stabilizes neuronal excitability and raises the seizure threshold (Begh et al. 2024; Lu and Wang 2015; Ríos et al. 2022).

Viniferin enhances the expression of brain-derived neurotrophic factor (BDNF), a key molecule in neuroplasticity and emotional regulation. By promoting synaptic connectivity, neurogenesis, and long-term potentiation, viniferin helps to reverse stress-induced cognitive deficits and improve mood regulation. Its ability to restore neurotrophin signaling may also have protective effects against stress-related neurodegeneration (Chen et al. 2022).

### Cardiovascular effect

Cardiovascular diseases (CVDs) remain the foremost cause of mortality worldwide, largely driven by oxidative stress, inflammation, endothelial dysfunction, and thrombosis. Viniferin, a resveratrol-derived oligomeric stilbenoid found primarily in *Vitis vinifera*, has garnered significant interest due to its multi-targeted biological activity. This section presents the current understanding of viniferin’s cardiovascular benefits, emphasizing mechanistic pathways and experimental evidence (Contreras et al. 2023).

Viniferin, particularly ε-viniferin, exhibits strong free radical-scavenging properties attributed to its multiple hydroxyl groups and polymeric structure. Research has shown that viniferin increases the expression and activity of key antioxidant enzymes, including SOD, CAT, and GPx, all of which are essential in neutralizing excess ROS (Fernandes et al. 2021). Simultaneously, viniferin significantly reduces MDA concentrations, a marker of oxidative lipid damage, in vascular tissues. This dual role in enhancing antioxidant defenses and suppressing pro-oxidant byproducts contributes to improved vascular health and reduced risk of oxidative injury in cardiovascular systems (Sy 2023; Wu et al. 2020a; Zghonda 2012).

Inflammation plays a central role in endothelial dysfunction, plaque formation, and myocardial damage (Higashi 2022; Medina-Leyte et al. 2021). Viniferin demonstrates potent anti-inflammatory properties by targeting major inflammatory pathways. It inhibits the activation of NF-κB, a transcription factor responsible for the expression of numerous pro-inflammatory mediators. As a result, viniferin decreases levels of TNF-α, IL-1β, IL-6, and C-reactive protein (CRP), which are commonly elevated in patients with cardiovascular disease. Furthermore, viniferin downregulates leukocyte adhesion to the endothelium and prevents the infiltration of inflammatory cells into vascular tissues. These combined actions suggest that viniferin could effectively modulate vascular inflammation and stop the progression of atherosclerotic changes (Hung et al. 2019; Wu et al. 2020; Zghonda et al. 2011).

A properly functioning endothelium is critical for vascular homeostasis, as it regulates vasodilation, blood flow, and platelet activity. Endothelial dysfunction is closely linked to hypertension, which further accelerates cardiovascular complications (Drożdż et al. 2023; Xu et al. 2021). Viniferin has been shown to enhance eNOS expression, leading to increased production of NO, a key vasodilator. By improving NO availability, viniferin promotes endothelium-dependent relaxation and reduces arterial stiffness (Beaumont et al. 2022; Fernandez-Cruz et al. 2019). Additionally, viniferin inhibits calcium influx in vascular smooth muscle cells, facilitating vasodilation. In experimental models of hypertension, viniferin administration led to significant decreases in systolic and diastolic blood pressure. These effects were accompanied by improvements in vascular reactivity and reductions in oxidative stress markers, indicating that viniferin may help restore endothelial function and improve overall vascular tone through multiple pathways (Vinet et al. 2016; Wu et al. 2020).

One of viniferin’s critical anti-atherogenic mechanisms involves the inhibition of low-density lipoprotein (LDL) oxidation, a key event in the initiation of foam cell formation and plaque development. Viniferin also suppresses the proliferation and migration of vascular smooth muscle cells (VSMCs), which are involved in plaque growth and stability. Furthermore, viniferin improves lipid metabolism by lowering circulating levels of total cholesterol, LDL cholesterol, and triglycerides, while concurrently increasing levels of protective high-density lipoprotein (HDL) (Łanoszka and Vlčková 2023; Mingwei 2019; Wu et al. 2020).

Animal studies have demonstrated that chronic viniferin administration significantly reduces atherosclerotic plaque burden and improves histological features of arterial walls. These findings indicate a potential role for viniferin in both the prevention and regression of atherosclerotic lesions (Lu et al. 2017; Zghonda et al. 2012).

Viniferin exhibits a spectrum of cardioprotective actions in the setting of ischemia–reperfusion (I/R) injury. It reduces infarct size and myocardial necrosis, preserves mitochondrial integrity, and attenuates oxidative bursts associated with reperfusion. Moreover, viniferin helps maintain intracellular calcium balance, which is critical for cardiomyocyte survival. These effects translate to improved cardiac function and reduced structural damage, suggesting that viniferin may serve as a protective agent during ischemic events and cardiac surgeries (Peng et al. 2025; Seya et al. 2009; Vinet et al. 2016).

### Weight loss effect

Overweight and obesity have emerged as major public health concerns due to their rising prevalence across both developed and developing nations. Estimated 764 million adults and 157 million children aged 5 to 19 were classified as obese in 2020. Projections from the World Obesity Federation indicate that by 2030, obesity will affect approximately 20% of women and 14% of men globally (Ruze et al. 2023). These conditions, particularly obesity, not only compromise physical and mental health but also significantly increase the risk of numerous comorbidities, including hypertension, diabetes mellitus type 2, cardiovascular and respiratory diseases, and various forms of cancer (Bhoyrul and Lashock 2008).

Since these conditions stem from a sustained imbalance between energy intake and expenditure, the primary approach to treatment emphasizes lifestyle changes specifically, reducing calorie consumption and increasing physical activity to promote a negative energy balance (Gomez-Zorita et al. 2023). However, maintaining long-term adherence to such interventions remains challenging. While pharmacological options exist, their use is constrained by a limited number of approved drugs and associated side effects (Kokkorakis et al. 2025). Consequently, growing scientific attention has focused on exploring natural bioactive compounds found in foods and plants for their anti-obesity effects, highlighting their potential as supportive tools in preventing and treating excessive accumulation of body fat.

Amidst the rising interest in phytochemicals for obesity intervention, viniferin has emerged as a bioactive compound with notable anti-obesity potential. In vitro research has provided compelling evidence for the anti-adipogenic and anti-inflammatory properties of ε-viniferin. In a study conducted by Ohara et al. (2015), murine 3T3-L1 pre-adipocytes were treated throughout their 8-day differentiation period with ε-viniferin at concentrations of 12.5, 25, or 50 µM (Ohara et al. 2015). Notably, these effects were compared directly with those of trans-resveratrol which is its monomeric counterpart. ε-Viniferin significantly reduced triglyceride accumulation at 25 and 50 µM by 37% and 72%, respectively, whereas trans-resveratrol only showed a significant reduction at the highest concentration tested (50 µM).

Interestingly, despite its pronounced effect on lipid accumulation, ε-viniferin only downregulated the expression of peroxisome proliferator-activated receptor gamma (PPARγ), a central transcription factor in adipogenesis, at the highest dose, suggesting that additional mechanisms may contribute to its lipid-lowering effect. Since obesity is often associated with low-grade inflammation in adipose tissue, this research group also assessed the expression of monocyte chemoattractant protein-1 (MCP-1), a marker of macrophage infiltration and inflammation. ε-Viniferin significantly decreased MCP-1 expression at 25 and 50 µM, indicating a likely anti-inflammatory action in differentiating adipocytes (Ohara et al. 2015).

Additionally, the effects of ε-viniferin on mature adipocytes were also investigated. In the same 2015 study by Ohara et al., mature 3T3-L1 adipocytes on day 8 of differentiation were exposed to 50 µM of ε-viniferin for 24 h. While triglyceride content was not directly assessed in this experiment, MCP-1 mRNA levels were again found to be reduced, while PPARγ expression remained unchanged. These results align with those observed in pre-adipocytes and suggest that ε-viniferin exerts anti-inflammatory effects in both developing and mature adipocytes (Ohara et al. 2015). In 2017, Lu et al. examined lower concentrations of ε-viniferin (2.5, 5, and 10 µM) and found a dose-dependent inhibition of lipid accumulation in vitro, with triglyceride levels reduced to 96%, 93%, and 92% relative to control, respectively, confirming its efficacy even at modest doses and reinforcing its role in suppressing adipogenesis (Lu 2017).

Regarding in vivo models, all studies to date have used mouse models to explore the systemic effects of ε-viniferin. In the aforementioned study by Ohara et al. (2015), 5-week-old male C57BL/6J mice were administered high-fat diet (60% of energy from fat) for 4 weeks, with or without supplementation of 0.2% ε-viniferin. Mice receiving ε-viniferin showed significantly less weight gain, which was attributed in part to reductions in subcutaneous, epididymal, and retroperitoneal white adipose tissue masses. Interestingly, no significant effect was observed on the mesenteric fat depot. On the molecular level, ε-viniferin supplementation led to decreased mRNA expression of inflammatory markers such as MCP-1 and TNF-α in epididymal adipose tissue, confirming its anti-inflammatory action previously observed in vitro (Ohara et al. 2015). Also, ε-viniferin reduced both the gene expression and circulating levels of leptin, a hormone that is produced by adipose tissue and helps regulate energy balance by suppressing appetite and increasing energy expenditure (Pico et al. 2022), though plasma MCP-1 levels remained unchanged, suggesting that some systemic inflammatory pathways may not be equally responsive.

In the study of Lu (2017), these researchers used the same mouse strain, where animals were divided into three groups: one receiving a standard diet, one fed a high-fat diet, and another fed a high-fat diet alongside oral treatment with (+)-ε-viniferin. The viniferin-treated group received 10 mg/kg/day for the first 38 days, then 25 mg/kg/day for the remaining 20 days of the 58-day study. Compared to the untreated high-fat-fed mice, those given ε-viniferin gained significantly less weight and showed 3-hydroxy-3-methyl glutaryl-CoA reductase activity inhibition, which is a key enzyme for catalyzing cholesterol synthesis (Lu 2017).

However, their body weights remained higher than those of mice on a standard diet, suggesting a partial but meaningful effect in mitigating diet-induced obesity. In contrast to Ohara et al.’s earlier findings, where multiple fat depots were reduced, this study observed a significant reduction only in the mesenteric adipose tissue (Lu 2017). Unfortunately, the molecular mechanisms underlying this depot-specific effect have not been explored in the follow-up work, highlighting an area for future investigation.

Building upon the earlier in vitro and in vivo findings, a study by Hung et al. (2019) provided deeper insights into the comparative efficacy of ε-viniferin and resveratrol in modulating adipogenesis and lipid metabolism in 3T3-L1 pre-adipocytes. This study revealed that ε-viniferin was significantly more potent than resveratrol in suppressing intracellular lipid accumulation, even at non-toxic concentrations that do not induce apoptosis. While both compounds belong to the polyphenolic family and are abundant in red wine, ε-viniferin demonstrated distinct biological actions that resveratrol did not replicate. Specifically, ε-viniferin markedly downregulated the key transcription factors PPARγ and CCAAT/enhancer-binding protein alpha (C/EBPα), as well as downstream metabolic enzymes like fatty acid synthase (FAS), the pivotal regulators of adipocyte differentiation and lipogenesis (Li et al. 2016b; Song et al. 2017; Wang et al. 2004). At the same time, it enhanced the expression of adipose triglyceride lipase (ATGL), a key enzyme in lipolysis (Zimmermann 2009), effectively promoting lipid breakdown over storage. Moreover, ε-viniferin uniquely upregulated the expression of adiponectin, an adipokine known for its anti-inflammatory, insulin-sensitizing, and lipid-regulating roles. This is particularly significant, as reduced adiponectin levels are associated with insulin resistance, T2DM, and metabolic syndrome (Li, et al. 2024).

The study also revealed that ε-viniferin activated important metabolic sensors: it increased the phosphorylation of p-AMPK and elevated levels of SIRT1, a NAD⁺-dependent deacetylase that functions synergistically with AMPK to inhibit PPARγ and C/EBPα activity and improve mitochondrial function (Jiang et al.^2012^; Picard et al. 2004). These coordinated molecular effects suggest that ε-viniferin fosters a healthier form of adipocyte differentiation, thus enhances the number of insulin-sensitive, adiponectin-producing cells while minimizing lipid storage (Hung et al. 2019). Hence, ε-viniferin’s ability to modulate adipocyte behavior at multiple levels: suppressing lipogenesis, promoting lipolysis, enhancing beneficial adipokines, and activating metabolic pathways makes it a multifaceted and promising phytochemical candidate in the dietary management of obesity and related metabolic disorders.

### Gastrointestinal effect

In the intestinal lining, two key chloride (Cl⁻) channels play major roles in fluid secretion and are closely linked to various types of diarrheas. The first, known as the cystic fibrosis transmembrane conductance regulator (CFTR), is activated by increased levels of cyclic nucleotides and is responsible for the excessive Cl⁻ and water secretion seen in conditions like cholera and traveler’s diarrhea, often triggered by enterotoxigenic *E. coli* (Rivera-Chávez 2022). The second channel, called the calcium-activated chloride channel (CaCC), becomes active when there’s a rise in intracellular calcium levels. This channel is particularly involved in rotavirus-induced diarrhea and is also believed to contribute to diarrhea caused by certain medications and potentially other viruses (Morris 1999).

These channels are involved in Cl⁻ secretion into the gut lumen, a process central to fluid movement and diarrheal pathophysiology, and their dysregulation is often implicated in both infectious and functional diarrheal states.

Viniferin has emerged as a promising therapeutic agent against diarrhea, particularly secretory diarrheal disorders and irritable bowel syndrome with diarrhea predominance (IBS-D). Among its active forms, trans-ε-viniferin and trans-δ-viniferin have demonstrated robust antisecretory and antispasmodic activities that are mechanistically linked to their inhibition of CFTR and CaCCs, especially TMEM16A, the native CaCC expressed abundantly in intestinal epithelial cells and interstitial cells of Cajal (ICCs) (Huang 2009; Hwang et al. 2009).

In 2018, a group of Chinese researchers reported that trans-ε-viniferin significantly inhibits CaCC current in HT-29 human colonic cells and in mouse colon tissue with an IC₅₀ near 1 μM. In a neonatal mouse model of rotavirus-induced secretory diarrhea, oral administration of trans-ε-viniferin resulted in a greater than 50% reduction in stool water content without affecting viral load, indicating its direct influence on epithelial secretion rather than viral replication. These findings were supported by electrophysiological assays using Ussing chambers that demonstrated viniferin’s potent ability to inhibit ATP-stimulated Cl⁻ secretion across the colonic epithelium, suggesting a pharmacologically relevant suppression of CaCC-mediated ion transport. Notably, the inhibition did not interfere with cytosolic Ca^2^⁺ signaling, reinforcing the specificity of viniferin’s action on channel gating rather than upstream calcium mobilization (Yu et al. 2018).

In 2019, this research group confirmed that trans-δ-viniferin also exerts inhibitory effects on TMEM16A, with IC₅₀ values of 4.65 μM in HT-29 cells and 19.7 μM in Fischer rat thyroid (FRT) cells expressing TMEM16A, further extending viniferin’s antisecretory profile. Also, in rotavirus and IBS-D mouse models, TVN treatment not only reduced stool water content and diarrhea severity but also suppressed intestinal motility by decreasing smooth muscle contractility, an effect that was attributed to its inhibitory action on TMEM16A within the gastrointestinal muscle layers and ICCs. Moreover, trans-δ-viniferin has been shown to reduce Ca^2^⁺-activated K⁺ channel activity and intracellular calcium levels to some extent, which may complement its Cl⁻-suppressing effects by reducing overall epithelial excitability and muscle hypercontractility. These results suggest that viniferin regulates intestinal smooth muscle rhythm and fluid transport in a dual mechanism, acting as both a Cl⁻ channel blocker and a modulator of gut motility (Yu et al. 2019).

Recently, a report published in 2023 further substantiated these roles, demonstrating that trans-ε-viniferin inhibits intestinal TMEM16A with no effect on the intracytoplasmic calcium concentration or protein expression of TMEM16A (Liu et al. 2023).

Thus, viniferin has shown potent antidiarrheal potential which warrants its advancement into clinical studies for diarrheal diseases, especially those involving excessive Cl⁻-driven secretion and dysmotility, such as rotavirus enteritis and IBS-D.

### Anti-cancer and immunomodulatory activities

**Fig. 8:**
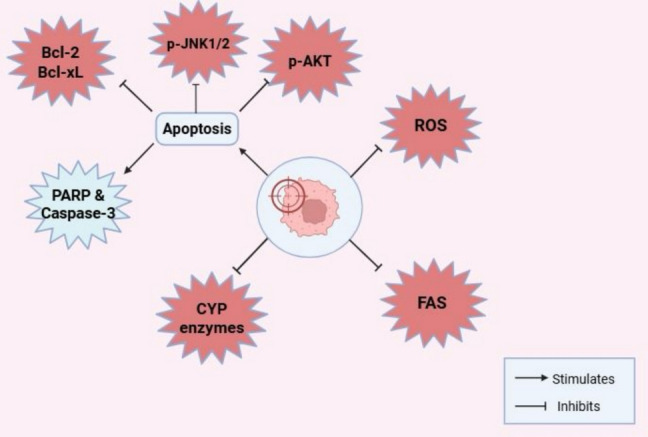
Anticancer effects of viniferin by various pathways. Bax, Bcl-2-associated X protein; Bcl-2, B cell lymphoma 2; Bcl-xL, B cell lymphoma-extra large; CYP, cytochrome P450; Fas, an apoptosis-related cell surface receptor; PARP, poly(ADP-ribose) polymerase; p-Akt, phosphorylated protein kinase B; p-JNK1/2, phosphorylated c-Jun N-terminal kinases 1 and 2; ROS, reactive oxygen species

Viniferin is an anticancer which is acting by various pathways (Fig. 8). It is present in many oligomeric forms, but the most active forms as anticancer are α, ε-viniferins, and r-viniferin. The activity of α and ε-viniferins was examined on non-small cell lung cancer cell line A549, melanoma cell line A2058, and osteosarcoma cell lines HOS and U2OS in a recent study (Huang et al. 2021).

The results showed that apoptosis in HOS cells was triggered by α-viniferin by 2 mechanisms. It first decreased the expression of phospho-c-Jun-N-terminal kinase 1/2 (p-JNK1/2), and the second mechanism is by increased expression of cleaved Poly (ADP-ribose) polymerase (PARP). ε-Viniferin was active as an antiproliferative agent in HOS cells, but α-viniferin was more effective. In A549 cells, α and ε-viniferin combination induced apoptosis by decreasing phospho-protein kinase B (p-AKT) expression and increasing cleaved PARP and cleaved caspase-3 expression (Huang et al. 2021). Moreover, in lymphoid and myeloid cell lines, antiproliferative activity was shown by viniferin through caspase-induced apoptosis (Barjot 2007). In addition, r-viniferin had prominent cytotoxic effects on prostate cancer cell lines, LNCaP, at low concentrations (Empl 2015).

Moreover, the antioxidant activity of viniferin contributes to its anticancer effect. Indeed, it is a free radical scavenger, hence inhibiting the oxidative damage induced by chemical carcinogens. In addition, viniferin is involved in inhibiting CYP enzymes. CYP enzymes are involved in xenobiotics metabolism and activation of some chemical carcinogens. Thus, inhibiting CYP enzymes by viniferin can contribute significantly to cancer prevention (Xue 2014).

In addition, vitisin B, which is a derivative of the viniferin, exhibited an anti-cancer effect especially in breast cancer. In breast cancer cells, the activity of FAS was significantly elevated. Noteworthy, fatty acids are utilized by cancer cells for proliferation. Interestingly, vitisin B was found to have FAS inhibitory activity, thus exerting an anticancer activity in breast cancer (Wang et al. 2019).

Another derivative of viniferin, namely, vitisin A, was studied in combination with TNF-related apoptosis-inducing ligand (TRAIL) in prostate cancer cell lines. This combination induced an anticancer effect via numerous mechanisms, like activation of procaspase 7/8, which eventually resulted in apoptosis. Additionally, combining vitisin A with TRAIL led to downregulation of the anti-apoptotic proteins Bcl-2 and Bcl-xL (Shin 2015).

In a cell-cycle specific manner, ε-viniferin arrests the cell cycle at G2/M phase (Tarhan et al. 2016). Fascinatingly, the acetylated form of ε-viniferin is more potent than the non-acetylated analog (Xue, et al. 2014). On the other hand, α-viniferin aborts the cell cycle mainly during the S phase. The α-viniferin exerts a strong anticancer effect in myeloid leukemia cell line, like HL-60 (Wibowo 2011).

Additionally, it showed a marked antiproliferative action against submandibular gland carcinoma (Chowdhury et al. 2005). Regarding vitisin A, it specifically aborts the cell cycle at G1 phase (Shin 2015).

### Antibacterial activity

The antibacterial action of α-viniferin is demonstrated against both drug-sensitive and drug-resistant *Mycobacterium tuberculosis* strains. Additionally, it demonstrates outstanding anti-Staphylococcus activity against three species of Staphylococcus, including methicillin-resistant *S. aureus* (MRSA), methicillin-susceptible *S. aureus* (MSSA), and methicillin-resistant *Staphylococcus epidermidis* (MRSE) (Seo et al. 2017). Prior research utilizing animal models has shown that α-viniferin enhances overall health in mammals and is quickly absorbed into the bloodstream (Fan et al. 2020). The main mechanisms of antibacterial activity are as follows:

- Disruption of bacterial membranes: by interacting with bacterial cell membranes, viniferin causes an increase in permeability and, ultimately, cell death. Certain Gram-positive bacteria, such *Staphylococcus aureus*, are more susceptible to this mechanism’s effects (Mattio 2019a).
- Inhibition of essential enzymes: viniferin derivatives demonstrate the ability to inhibit crucial bacterial enzymes, thereby disrupting metabolic processes vital for survival. For example, trans-δ-viniferin derivatives demonstrate significant antibacterial activity against *S. aureus* by focusing on enzymatic pathways (Huber 2023).
- Structure–activity relationships (SAR): research has demonstrated that particular chemical alterations, including O-methylation and halogenation, significantly augment the antibacterial efficacy of viniferin derivatives. These alterations enhance the compound’s capacity to engage with bacterial targets (Huber 2023).

### Antiviral activity

Viniferin is effective against a wide variety of enveloped viruses, including herpes simplex virus type 2 (HSV-2), SARS-CoV-2, and influenza virus. The mechanisms consist of:

- Direct virucidal effects: viniferin derivatives eliminate the ability of viral particles to enter host cells by rendering them inactive. Influenza viruses are especially susceptible to this mechanism’s efficacy (Zwygart et al. 2023).
- Cell-mediated effects: viniferin inhibits viral replication and spread by enhancing host cell-mediated responses. This process is demonstrated by the SARS-CoV-2 Delta strain (Zwygart et al. 2023).
- Inhibition of viral enzymes: viniferin interferes with the reproduction of viruses by targeting viral enzymes such the HCV NS3 helicase. Its ability to inhibit HCV replication relies on this process (Lee 2019).
- Prevention of biofilm formation: by inhibiting the development of viral biofilms, viniferin derivatives shorten the duration of viral infections (Yadav et al. 2019).

### Antiparasitic activity

Viniferin exhibits antiparasitic efficacy predominantly against helminths and protozoa. The mechanisms include:

- Tegumental damage: viniferin disrupts the arrangement of muscle bundles and disintegrates microtriches in helminths by inflicting severe structural damage to the tegument. This leads to the parasite’s inability to move and eventually its death (Giri and Roy 2015; Roy and Giri 2015a).
- Enzyme inhibition: viniferin acts as an inhibitor of essential tegumental enzymes, including adenosine triphosphatase, acid phosphatase, alkaline phosphatase, and nucleotidases, thereby disrupting energy production and nutrient metabolism. This disturbance impedes the parasite’s ability to survive and carry out its metabolic functions (Roy and Giri 2015a).
- Neurotransmitter modulation: viniferin influences enzymes associated with neurotransmitters, including nitric oxide synthase, and acetylcholinesterase, which are essential for neuromuscular communication in parasites. This modulation results in the paralysis and subsequent death of the parasite (Giri and Roy 2015).

### Antiplasmodial activity

People can contract malaria, a potentially deadly illness, from parasite species. *Plasmodium falciparum* and *Plasmodium vivax* are the most dangerous of all. More than half of the world’s population was reportedly at risk of catching malaria in 2020 (Zekar and Sharman 2025).

Medicinal herbs have been used in traditional medicines as part of the many attempts to prevent and treat malaria. The World Health Organization (WHO) has recommended preventive measures for addressing the condition via antimalarial pharmaceuticals. A multitude of studies examine suitable phytochemicals for health therapies.

The antiplasmodiac properties of viniferin have been discovered to be promising. The structure of viniferin was ascertained through their comprehensive spectrum analyses, which encompassed 1D, 2D, and infrared NMR. Alpha-glucosidase and alpha-amylase were inhibited by it, with 50% inhibitory concentrations (IC50) of 256.17 and 212.79 g/mL, respectively (Lulan 2020).

The inhibitory action against *Plasmodium falciparum* strain 3D7 was tested in vitro at a dose of 100 µg/mL, and the results showed a substantial antiplasmodial effect with an IC50 value of 2.76 µg/mL. The results suggest that viniferin, an isolated extract from *Dipterocarpus littoralis*, could be developed into an antiplasmodial agent (Lulan 2020). The mechanisms include:

- Inhibition of parasite growth: by focusing on vital metabolic pathways, viniferin derivatives impede the development of Plasmodium parasites. The capacity of the chemical to disrupt the parasite’s life cycle is the reason for this action.
- Antioxidant effects: viniferin’s antiplasmodial effectiveness is further enhanced by its antioxidant qualities, which lessen oxidative stress brought on by parasite infections (Fuloria et al. 2022a).

### Anthelmintic effects

Viniferin has been studied for its possible anthelmintic properties. Roy and Giri (2015a) reported that viniferin is a potent chemical in the plant *Carex baccans* L., which has anti-inflammatory, anticancer, and anti-diabetic effects. Historically, numerous communities in Northeast India have employed *C. baccans* as a therapy for intestinal worm diseases. A variety of viniferin concentrations were tested in vitro, including 50, 100, and 200 M/mL in physiological buffered saline, to assess helminth motility and mortality. Important tegumental enzymes, including adenosine triphosphatase, acid phosphatase, and alkaline phosphatase, exhibited decreased activity in parasites exposed to viniferin, according to histochemical and biochemical investigations. The compound’s cestocidal efficacy is demonstrated by the significant structural and functional changes seen in the treated parasites.

**Fig. 9:**
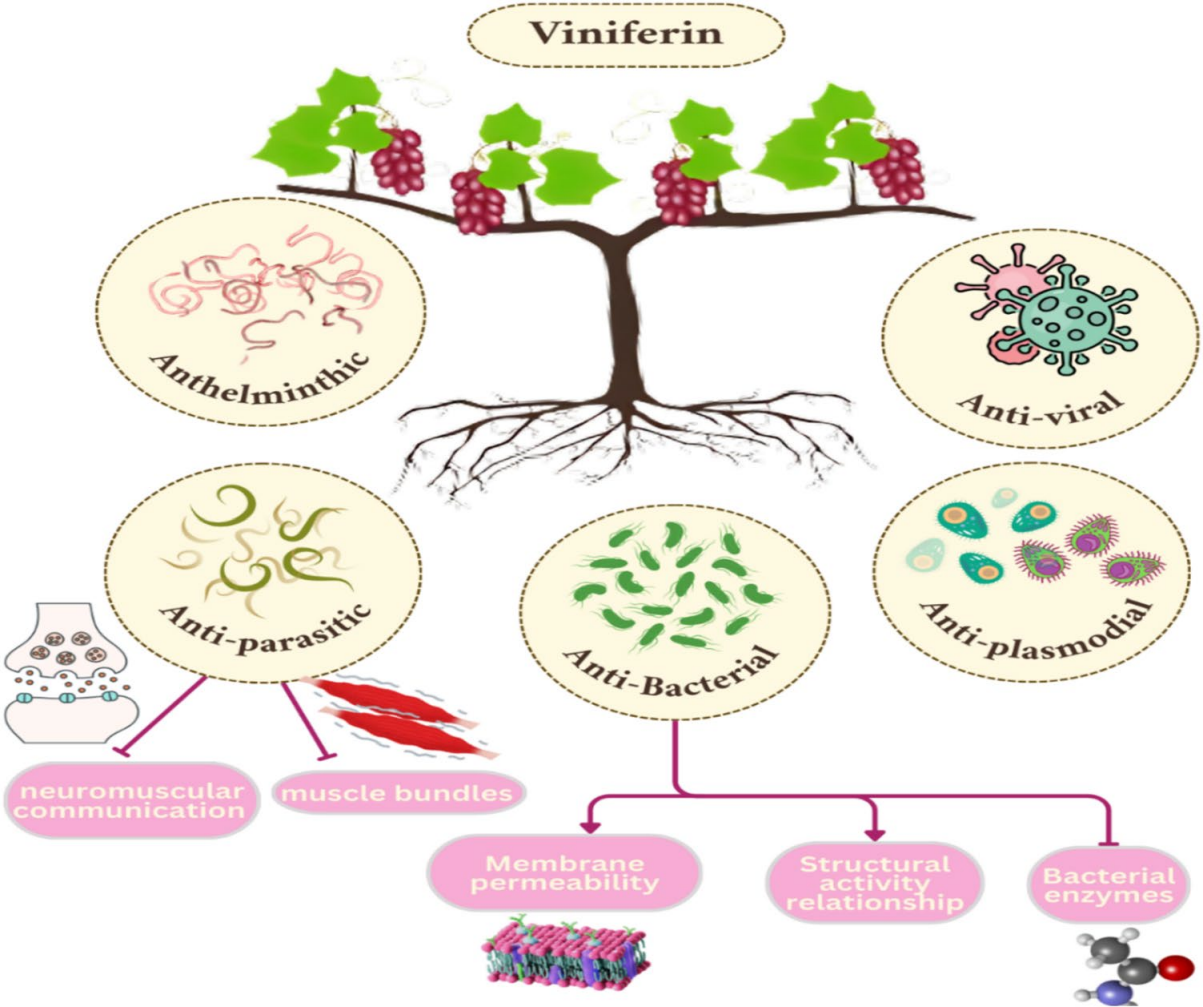
Antimicrobial, antiplasmodial, anthelmintic, and antiparasitic effects of viniferin

Resveratrol is responsible for the distortion and dying of suckers, which result in an increase in the anthelmintic effects of *Raillietina echinobothrida*. Resveratrol and viniferin are two polyphenols that have been shown to have the potential to have anthelmintic effects on *R. echinobothrida*. This is due to the fact that these polyphenols modify the NOS and AChE activities of the organism (Giri and Roy 2015). Finally, viniferin exhibits a wide range of biological activities, including antibacterial, antiparasitic, anthelmintic, antiplasmodial, and antiviral effects as illustrated in Fig. 9. These activities are mediated through various molecular mechanisms, which are discussed below.

### Cosmetic purposes: skin and hair disorders

**Fig. 10:**
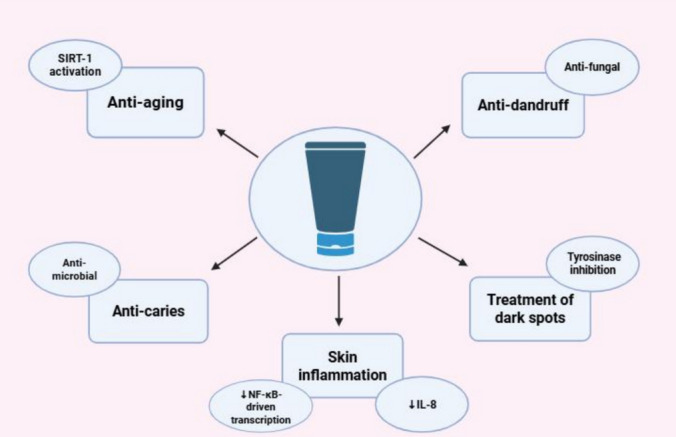
Viniferins cosmetic effects. IL-8, interleukin-8; NF-κB, nuclear factor kappa-B; SIRT1, sirtuin 1

Viniferins are used in skin products due to their beneficial cosmetic effects such as anti-aging, anti-dandruff, anti-caries, anti-inflammatory, and treatment for dark spots (Fig. 10). Viniferins, especially the ε-viniferin, are mainly utilized due to their marked anti-aging effect as well as the skin whitening action. Interestingly, its anti-aging property was mainly attributed to the activation of SIRT1 (Anna Malinowska et al. 2020).

SIRT1 prolongs cell survival through stimulating p53 deacetylation, which mainly maintains the pluripotent state (Anna Malinowska et al. 2020; Ong and Ramasamy 2018).

Moreover, ε-viniferin is used to combat dark spots. This action is specifically linked to its ability to inhibit tyrosinase enzyme (Anna Malinowska et al. 2020). Noteworthy, tyrosinase enzyme is the first step in the conversion of tyrosine into melanin (Pavan et al. 2020). Furthermore, various *Vitis vinifera* extracts were tested for its anti-inflammatory activity. *V. vinifera* extract was found to inhibit the production of pro-inflammatory cytokines, mainly the IL-8, and inhibition of NF-κB-driven transcription. Indeed, this anti-inflammatory activity was exploited cosmetically to reduce skin irritation (Sangiovanni 2019).

Besides, viniferin is incorporated in cosmetic preparations as an anti-dandruff, and this is possibly due to its anti-fungal effect. In addition, it serves in oral care products as anti-caries because of its anti-microbial properties.

## Toxicological profiles of individual viniferins

- α-Viniferin: displays dose-dependent hepatotoxicity in vivo, causing liver cell death without cell cycle arrest in albino mice. Low oral bioavailability and rapid systemic clearance in rats further complicate its toxicological assessment.
- ε-Viniferin: exhibits dose-dependent cytotoxicity in intestinal (Caco-2) cells, which can be mitigated through encapsulation (e.g., multi-lamellar liposomes) (Courtois 2019). Extensive hepatic metabolism is observed, including sulfation and glucuronidation in humans and rats, with most metabolites concentrated in the liver (Courtois et al. 2018; Courtois et al. 2017).

Limited in vivo toxicological studies suggest moderate safety but incomplete long-term or chronic toxicity data (Courtois et al. 2018).

R- and R2-viniferins: both are highly cytotoxic in vitro, with R2-viniferin showing stronger potency in HepG2 cells with an IC50 of 9.7 µM, 3 × more potent than resveratrol (Aja et al. 2020), while R-viniferin induces G1 phase cell cycle arrest and apoptosis in LNCaP prostate cancer cells (Empl 2015). Their toxicological evaluation is predominantly limited to cancer cell lines, with no comprehensive data on systemic or non-cancer toxicity.

Differential toxicity appears linked to structural variations, influencing ROS modulation, apoptosis induction, and receptor interactions: α- and ε-viniferins inhibit epithelial-mesenchymal transition (EMT) and ROS-mediated mechanisms in cancer cells (Chiou 2022). Metabolic profiles suggest glucuronide and sulfate conjugates as potential toxicity modulators but lack further mechanistic clarity (Courtois et al. 2018b; Courtois et al. 2017). For β-, γ-, and δ-viniferin, no empirical toxicology results are available based on the current data and references.

## Clinical trials on α-viniferin

While in vitro studies highlight various biological activities of viniferin isomers, translating these findings into clinical applications requires rigorous testing in humans. Clinical data on viniferins remain relatively limited compared to their precursor, resveratrol. However, existing studies, primarily focusing on topical applications of α-viniferin, provide initial evidence for its efficacy and safety in specific contexts. Additionally, preclinical studies, particularly with ε-viniferin, explore potential benefits in areas like neurodegeneration.

Antimicrobial activity: nasal decolonization of *S. aureus*

A significant clinical application explored for α-viniferin is its potential as a selective antimicrobial agent. Rahim et al. 2021) conducted a 10-day clinical trial involving 20 healthy adult females to evaluate the efficacy of topical α-viniferin (100 µg/mL suspension) for nasal decolonization of *S. aureus* (Rahim, et al. 2021). The study demonstrated promising results:

o Efficacy: treatment led to a significant reduction in both MSSA and MRSA counts in the nares (*p* = 0.002 and *p* = 0.008, respectively), based on culture quantification (Rahim, et al. 2021).
o Molecular analysis (16S rRNA sequencing) confirmed a significant decrease in the relative abundance of the *Staphylococcus* genus (Rahim, et al. 2021).
o Selectivity: crucially, the treatment did not significantly alter the counts of commensal bacteria or the overall diversity and composition of the nasal microbiota. This indicates a selective action against *S. aureus* without disrupting the normal flora (Rahim, et al. 2021).
o Safety and tolerability: prior skin irritation patch tests showed α-viniferin had low irritation potential up to 1000 µg/mL. During the 10-day trial, no adverse events or skin irritation were reported. Furthermore, an unexpected finding was a significant increase in skin moisture content, suggesting a potential moisturizing benefit (Rahim, et al. 2021).

This trial provides clinical evidence for α-viniferin’s potential as a safe and effective topical agent for selectively targeting *S. aureus* nasal carriage, a significant factor in healthcare-associated infections.

Dermatological application: facial hyperpigmentation

Another area where α-viniferin has shown clinical promise is in dermatology, specifically for treating hyperpigmentation disorders like melasma and freckles. Yun et al. (2018) investigated the effects of a topical extract from *Caragana sinica*, containing α-viniferin as a standardized active constituent (Yun et al. 2018). Key findings include:

o Clinical efficacy: in a randomized, double-blind, vehicle-controlled, split-face trial involving 23 patients, topical application of the *C. sinica* extract resulted in a decreased melanin index on facial melasma and freckles (Yun et al. 2018).
o *In vitro* corroboration: the study complemented clinical findings with in vitro work showing that α-viniferin inhibited melanin production in various cAMP-elevated melanocyte models (stimulated by α-MSH, histamine, or db-cAMP), proving more potent than the standard whitening agent arbutin (Yun et al. 2018).
o Mechanism: the researchers proposed a mechanism involving the acceleration of PKA inactivation a feedback termination step in melanogenesis ultimately leading to reduced expression of key melanogenic factors like MITF-M and tyrosinase (Yun et al. 2018).
o Safety: the extract, at concentrations demonstrating antimelanogenic activity in vitro, did not exhibit cytotoxicity (Yun et al. 2018).

This study supports the use of α-viniferin (as part of an extract) as a topical treatment for improving facial hyperpigmentation, linking clinical observation with a plausible molecular mechanism.

## Preclinical evidence: ε-viniferin and neuroprotection

Beyond topical applications, preclinical research explores the potential systemic effects of other viniferin isomers. Freyssin et al. (2022) investigated trans ε-viniferin in an APPswePS1dE9 transgenic mouse model of AD (Freyssin et al. 2022). While this is *preclinical* data, it offers insights into potential neuroprotective roles:

o Model and administration: mice received weekly intraperitoneal injections of 20 mg/kg ε-viniferin (compared against resveratrol and vehicle) over several months (Freyssin et al. 2022).
o Amyloid pathology: ε-viniferin demonstrated greater efficiency than resveratrol in decreasing hippocampal amyloid load and deposits (Freyssin et al. 2022).
o Cognition: both ε-viniferin and resveratrol treatments partially prevented the cognitive decline observed in the AD mouse model, as assessed by the Morris water maze test (Freyssin et al. 2022).
o Neuroinflammation: ε-viniferin treatment led to a significant decrease in brain uptake of a PET tracer for neuroinflammation (TSPO tracer [18F] DPA-714) compared to resveratrol. It also reduced the expression of neuroinflammatory markers glial fibrillary acidic protein (GFAP), ionized calcium-binding adapter molecule 1 (IBA1), and IL-1β (Freyssin et al. 2022). A caveat noted was the potential confounding neuroinflammatory effect of the vehicle (PEG 200) used (Freyssin et al. 2022).

These preclinical findings suggest that ε-viniferin warrants further investigation for neurodegenerative conditions like AD, potentially offering advantages over resveratrol in targeting amyloid pathology and neuroinflammation. However, these results are from an animal model using IP injection and cannot be directly extrapolated to human clinical efficacy, especially considering the poor oral bioavailability discussed previously.

## Conclusion

Viniferin and its various forms exhibit a broad spectrum of bioactivities, anti-inflammatory, antidiabetic, anticancer, antioxidant, neuroprotective, antiviral, and antimicrobial positioning this resveratrol-derived natural product as a credible candidate for interventions spanning chronic inflammation, metabolic disorders, infections, and neurodegeneration. To translate this promise into clinically meaningful outcomes, the next phase of research should prioritize (1) pharmacokinetic optimization (improving oral bioavailability, solubility, and metabolic stability; defining absorption, distribution, metabolism, and excretion with human-relevant models; and exploring advanced delivery systems such as lipid-based carriers, nanoparticles, and prodrugs); (2) systematic structure–activity relationship (SAR) and analog development to enhance potency, selectivity, target engagement, and safety while addressing isomer-specific activity among α/β/ε-viniferins and oligomeric forms; (3) robust target deconvolution and mechanism-of-action studies across key pathways (e.g., NF-κB, AMPK, PI3K/AKT/mTOR, sirtuins) to guide rational medicinal chemistry and biomarker selection; (4) well-designed preclinical disease models with PK/PD integration and toxicity profiling (including cardiometabolic, oncology, infectious, and neurodegenerative indications), alongside drug–drug interaction risk assessment; and (5) staged clinical evaluation, beginning with standardized formulations and phase 0/1 trials for safety, tolerability, and exposure, progressing to phase 2 efficacy in prioritized indications with pharmacodynamic biomarkers and patient stratification strategies. In parallel, standardization of source materials, GMP manufacturing, and regulatory pathway definition (drug vs. nutraceutical/cosmeceutical) will be critical for quality, scalability, and global access. Success across these fronts could enable affordable, non-toxic, and widely accessible viniferin-based therapeutics and adjuncts while also accelerating applications in nutraceuticals, cosmeceuticals, and agriculture through validated formulations, stability data, and safety standards. This forward agenda provides a clear translational roadmap from bench to bedside for the diverse biological promise of viniferin.

## Future directions: addressing human safety and toxicity data gaps of viniferin and its derivatives

To ensure the safe development of viniferin and its derivatives, future research should focus on establishing a comprehensive toxicological profile through acute, chronic, and long-term exposure studies, with attention to organ-specific and cumulative effects as well as drug interactions. Mechanistic investigations into metabolism and cellular pathways are needed to identify potential toxic metabolites and clarify modes of toxicity. Early-phase clinical trials should assess safety and tolerability in both healthy volunteers and patient populations, supported by systematic monitoring of adverse events. Aligning safety data with regulatory standards, while engaging patients and the public to address perceptions and ensure transparent communication, will be essential to bridge preclinical findings with clinical application.

## Data Availability

All source data for this work (or generated in this study) are available upon reasonable request.
